# Global expert views on the diagnosis, classification and pharmacotherapy of allergic rhinitis in clinical practice using a modified Delphi panel technique

**DOI:** 10.1016/j.waojou.2023.100800

**Published:** 2023-07-17

**Authors:** Désirée ES. Larenas-Linnemann, José L. Mayorga-Butrón, Juan Maza-Solano, Alexander V. Emelyanov, Ricardo LL. Dolci, Marcel M. Miyake, Yoshitaka Okamoto

**Affiliations:** a Center of Excellence in Asthma and Allergy, Hospital Médica Sur, Mexico City, Mexico; b Otolaryngology Department, National Institute of Pediatrics, Mexico City, Mexico; c Rhinology/Skull Base Surgery Unit, Hospital Universitario Virgen Macarena, Seville, Spain; d North-Western State Medical University named after I.I. Mechnikov, St. Petersburg, Russia; e Department of Otolaryngology, Santa Casa de São Paulo School of Medical Sciences, São Paulo, Brazil; f Chiba Rosai Hospital, Ichihara, Japan; g Chiba University, Inage Ward, Chiba, Japan

**Keywords:** Allergic rhinitis, Delphi method, Physician questionnaire, Treatment, Diagnosis

## Abstract

**Background:**

Diagnosis, classification, and treatment of allergic rhinitis (AR) varies considerably despite the availability of treatment guidelines.

**Objectives:**

We aimed to carry out a two-part modified Delphi panel study to elucidate global expert management of AR in real life.

**Methods:**

The modified Delphi panel study was composed of two ten-minute online questionnaires sent to global AR experts, aiming to identify areas of consensus (defined as >75% respondent agreement) on aspects of their real-world daily practice related to AR diagnosis, classification, and pharmacotherapy. A workshop discussion with respondents held after the first-round questionnaire informed the development of the second-round questionnaire.

**Results:**

Eighteen experts (from 7 countries across 3 continents) completed both questionnaires in September–October 2021 and January 2022, respectively. The majority of respondents agreed that diagnosis of AR is best confirmed using a mixture of observation and testing (n = 15) and collaborating with colleagues across other specialties (n = 14). Experts agreed that severity (n = 18), upper/lower respiratory tract involvement (n = 15) and symptom frequency (n = 14) are important factors when classifying AR, however consensus was not reached on which classification tool should be used. Although there were mixed opinions on the preferred pharmacotherapy treatment in the presented case studies, respondents largely agreed on which treatments require less monitoring (intranasal corticosteroid therapies [INCS]) and when treatments should be stepped down (≤3 months). Although opinions varied across respondents, some respondents considered as-needed INCS treatment and surgery to be viable treatment options.

**Conclusion:**

We identified clear differences between real-world practice and treatment guidelines related to the management of AR. Furthermore, we recognized differences among physicians concerning their clinical practice in the pharmacological treatment of AR. These findings highlight the need for greater research into the management of AR and further indicate there is still a major gap between treatment guidelines and daily practice, even among specialists, suggesting a need for local guideline adaptation and implementation plans.

## Introduction

Allergic rhinitis (AR) is defined as a symptomatic nasal disorder caused by immunoglobulin E (IgE)-mediated inflammation of the membranes lining the nose after exposure to an allergen.^1^^,^^2^ Patients experience characteristic nasal symptoms such as nasal obstruction, rhinorrhoea, sneezing, and nasal pruritus/itching; often accompanied by ocular symptoms including pruritus and epiphora.^1^, ^2^, ^3^ AR is one of the most common chronic respiratory conditions, notably in high-income countries where prevalence is as high as 50%.^2^ In low- and middle-income countries, prevalence is relatively low but steadily increasing.^1^^,^^2^ Despite global treatment variation, patients with AR are largely treated by general practitioners (GPs) and specialists including ear, nose, and throat (ENT) specialists, allergists, and pulmonologists.^4^^,^^5^

As is the case across multiple other disease areas,^6^, ^7^, ^8^ high-quality, evidence-based treatment guidelines have been developed to provide recommendations both for classification and pharmacological and non-pharmacological treatment approaches for AR. Prominent AR treatment guidelines include the “European Forum for Research and Education in Allergy and Airway Diseases (EUFOREA) 2020” guideline,^9^^,^^10^ “Allergic Rhinitis and its Impact on Asthma (ARIA) 2008”^2^ and “ARIA 2020”^1^ guidelines, and the “Rhinitis 2020: A practice parameter update” guidelines.^3^ Other treatment guidelines have been published, including those developed for physicians in Britain,^11^ the United States,^12^ Japan,^13^ Mexico,^14^ and Brazil,^15^ to name a few. There are various different validated severity scores and scales^13^^,^^16^, ^17^, ^18^, ^19^ that have been used to classify AR and to assess treatment response. However, the real-world diagnosis, classification and treatment of AR vary considerably*.*

In this study, we explore daily practice in the context of AR among specialists in several parts of the world. To best capture the unbiased opinion from all specialists, we employed a modified Delphi panel study, in which a small group of practicing clinical experts were selected and invited to complete 2 consecutive, anonymous questionnaires. Groups in Delphi panel studies are often small, typically up to 25 respondents, in order to reach and achieve consensus among the panel.^20^, ^21^, ^22^ Where a greater number of respondents is used, a high degree of variation is likely to occur, potentially reducing accuracy and limiting the ability to reach a consensus.^21^ Delphi panel studies are designed to use sequential questionnaires, building upon responses obtained from previous rounds of questions, in order to achieve consensus among the panel. Here, we report the findings of a two-part Delphi panel study that was designed to elucidate how ENT specialists and allergy specialists from different countries diagnose, classify and treat AR.

## Methods

### Questionnaire development

The Delphi survey technique is a recognized group facilitation approach designed to transform opinion into group consensus.^23^ This modified Delphi panel study consisted of 2 anonymous, ten-minute online questionnaires with a mixture of multiple-choice and open-response questions on the diagnosis, classification, and pharmacotherapy of AR (Supplemental Appendix). The first questionnaire was disseminated to panellists in September 2021, and the second questionnaire in January 2022. Between the 2 questionnaires, an expert discussion panel was held (December 2021) where the authors analyzed and discussed the findings of the first-round questionnaire, rationalized the responses, and proposed questions for inclusion in the second-round questionnaire. The questionnaires were developed under lead author direction, and conducted by a research team at Ashfield MedComms, an Inizio company (funded by GSK); all questionnaire content was reviewed, revised, and approved by the lead author. Further details on the questionnaires are provided in the Supplemental Appendix.

### Panellists

All 7 authors (from Brazil, Japan, Mexico, Russia, and Spain) are practicing clinicians and participated in this modified Delphi panel study. Fourteen additional healthcare practitioners with a professional interest in AR were invited by GSK to complete the questionnaire, based on their experience in AR. In total, 18 practicing clinicians completed both questionnaires and were general practitioners or specialists including ENT specialists, allergists, pediatricians, pulmonologists, and hospital directors. Patients were not consulted during the study. All panellists provided written informed consent to participate.

### Analysis and interpretation of responses

Panellist responses to the first- and second-round questionnaires were compared in order to reach a consensus regarding challenges and approaches in the diagnosis, classification, and medical treatment of AR in the panellists’ own clinical practice. Descriptive summaries were produced for both questionnaires (Supplemental Appendix). Consensus on an item was reached when >75% of panellists voted in agreement for the respective question. Analysis of the data from the first questionnaire formed part of the expert panel discussion (December 2021). Further details on coding of responses are available in the Supplemental Appendix.

## Results

Overall, 18 experts from 7 countries (7 authors and 11/14 additional AR experts) completed both questionnaires. Full questionnaire data are included in the Supplemental Appendix.

## AR is diagnosed using a multi-faceted approach

The questionnaire responses indicate a multi-disciplinary approach is preferred by those surveyed for diagnosing AR.

**Fig. 1:**
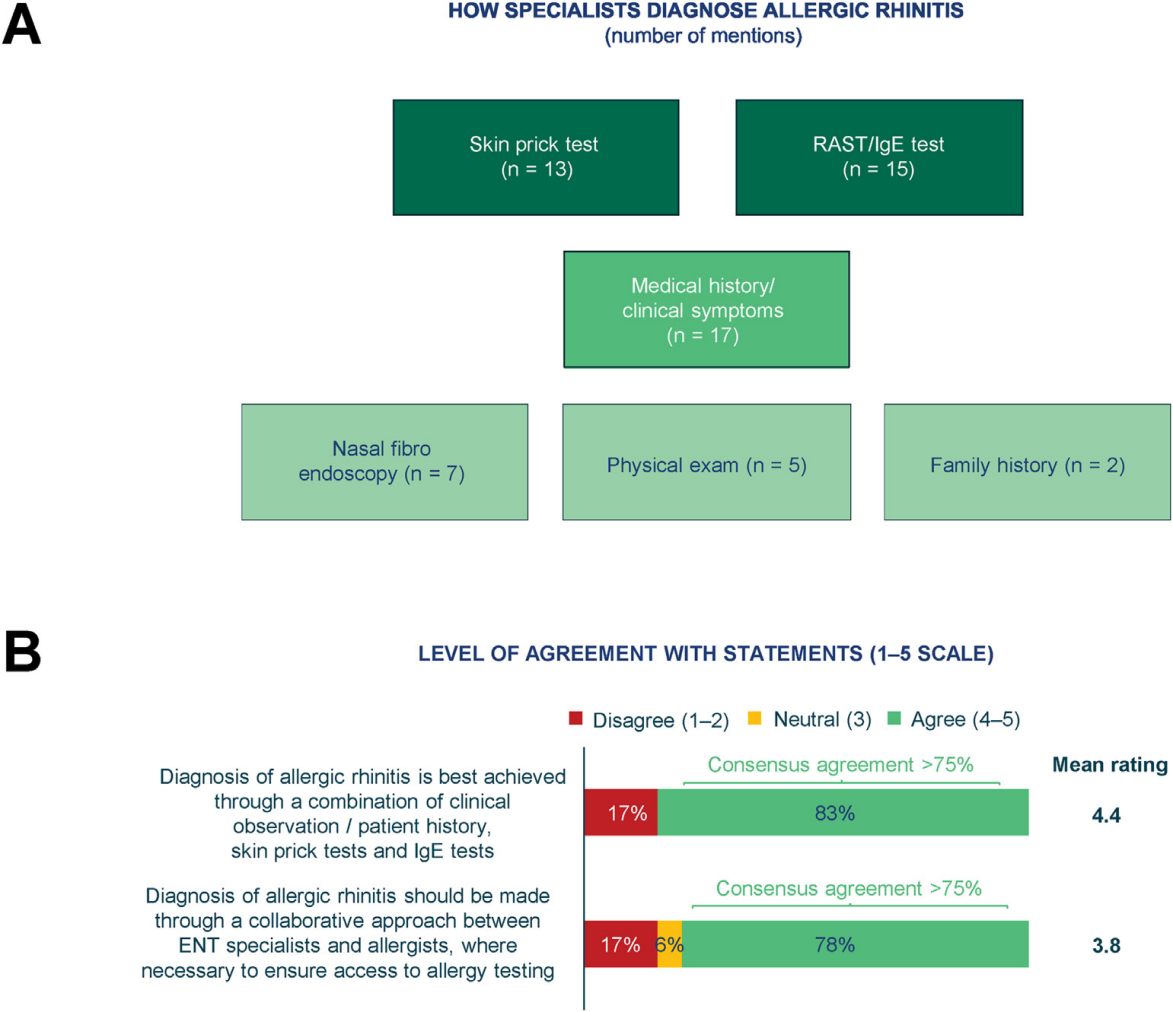
First-round (A) and second-round (B) questionnaire findings on diagnosis of AR. A: First-round questionnaire, Q1: How do you currently diagnose allergic rhinitis? Base: N = 18. Respondents could select multiple options. B: Second-round questionnaire, Q1: To what extent do you agree or disagree with the following statements? Diagnosis of allergic rhinitis is best achieved through a combination of clinical observation / patient history, skin prick tests and IgE tests. Diagnosis of allergic rhinitis should be made through a collaborative approach between ENT specialists and allergists, where necessary to ensure access to allergy testing. Base: N = 18. AR, allergic rhinitis; ENT, ear, nose and throat; IgE, immunoglobulin E; Q, question; RAST, radio-allergosorbent test serum specific IgE test

The aim of the questions regarding diagnostic methods from the first-round questionnaire was clarified during the panel discussion. In the subsequent round, equivalent questions were asked in the second-round questionnaire, and respondents agreed that the diagnosis of AR is best confirmed through a combination of clinical observation/patient history and the demonstration of specific IgE in skin testing or serum IgE tests. Thus, the majority of survey respondents (15/18) agreed that diagnosis is best confirmed through a combination of observation and testing (Fig. 1A), and, if necessary, there should be collaboration between ENT specialists and allergists to ensure access to allergy testing (14/18 respondents, mainly the non-allergists based on the expert panel discussion) (Fig. 1B).

### AR is classified using a range of severity scales/tools

**Fig. 2:**
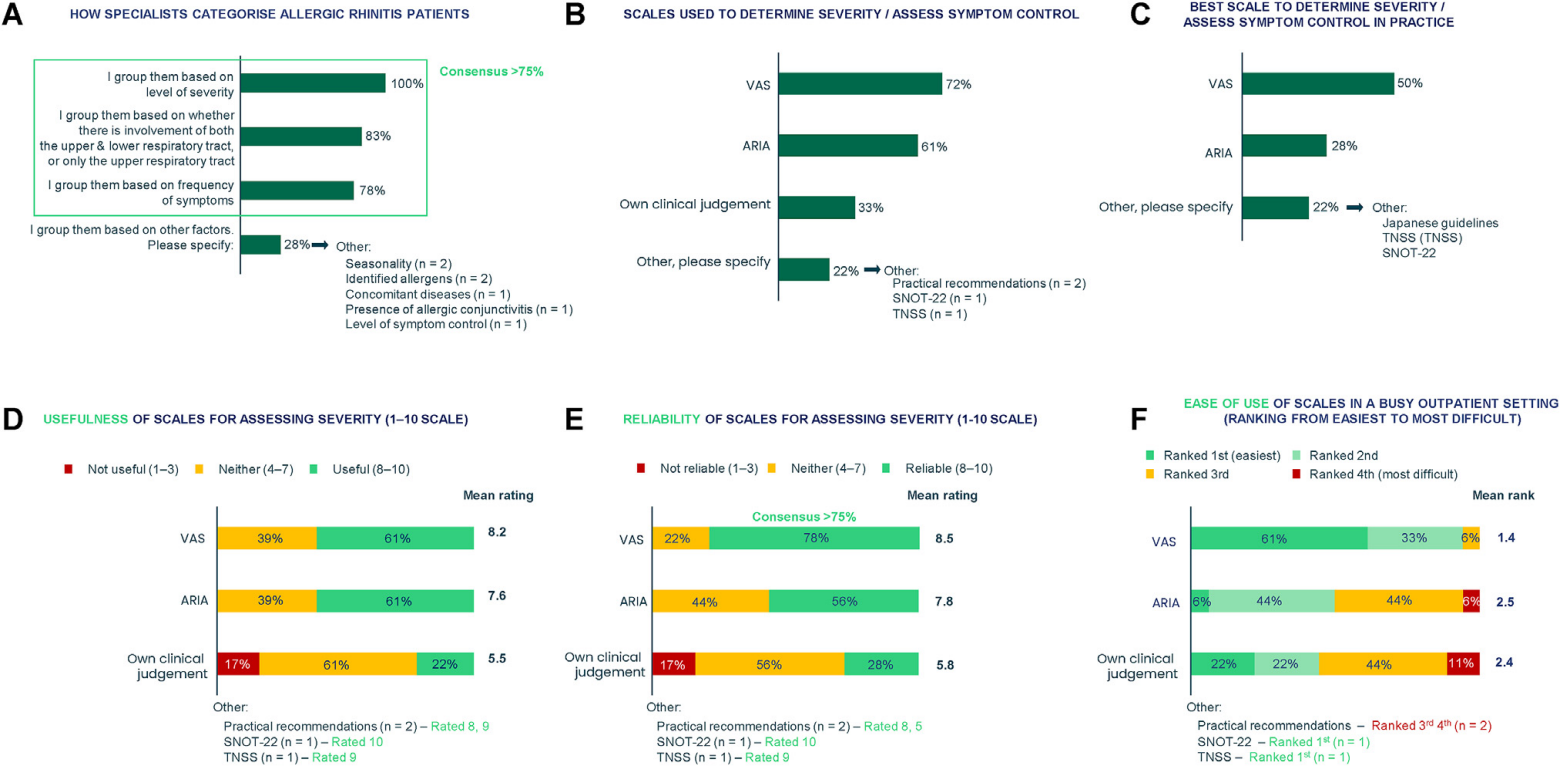
First-round (A, B, D–F) and second-round (C) questionnaire findings on classification of AR and comparison of severity scales. A: First-round questionnaire, Q2: Once a patient has been diagnosed with allergic rhinitis, how do you group / categorise them? Base: N = 18. Respondents could select multiple options. B. First round-questionnaire, Q3: Please think about how you assess the severity and/or symptom control of a patient diagnosed with allergic rhinitis. Taking into account all factors, including usefulness, reliability and ease of use, which scale do you think is the best assessment to use in clinical practice? Base: N = 18. C: Second-round questionnaire, Q2: What scales do you use to determine allergic rhinitis severity (mild/moderate/severe) and/or the assessment of symptom control? Base: N = 18. Respondents could select multiple options. D: First-round questionnaire, Q4: How useful do you find these scales for assessing severity of allergic rhinitis? Please rate on a scale of 1–10, where 1 is not at all useful and 10 is extremely useful. Base: N = 18. E: First-round questionnaire, Q5: How reliable do you find these scales for assessing severity of allergic rhinitis? Please rate on a scale of 1–10, where 1 is not at all useful and 10 is extremely useful. Base: N = 18. F: First-round questionnaire, Q6: Please rank these scales in order from the easiest to use to the most difficult to use in a busy outpatient setting. Base: N = 18. AR, allergic rhinitis; ARIA, Allergic Rhinitis and its Impact on Asthma; Q, question; SNOT-22, sino-nasal outcome test; TNSS, total nasal symptoms score; VAS, visual analogue scale

Respondents were aligned on the factors used to classify AR (Fig. 2A) but use different tools to do so (Fig. 2B–F). Experts returned decisive responses indicating severity (18/18 respondents), involvement of the upper/lower respiratory tract (15/18 respondents) and frequency of symptoms (14/18 respondents) as being most important when classifying AR (Fig. 2A).

When queried on the choice between the Visual Analogue Scale (VAS)^18^ and the 2008 ARIA guidelines’ AR severity classification,^1^^,^^2^ VAS was the most used to determine severity or assess symptom control (13/18 respondents) (Fig. 2B). There was no decisive preference for which assessment scale is best to use (Fig. 2C), the most useful (Fig. 2D), or the easiest to use (Fig. 2F); however, the VAS was favoured by half of those surveyed (9/18 respondents [Fig. 2C]). There was consensus that the VAS was deemed the most reliable scale for assessing severity – 14/18 participants rated the VAS at least 8 out of 10 (Fig. 2E).

**Fig. 3:**
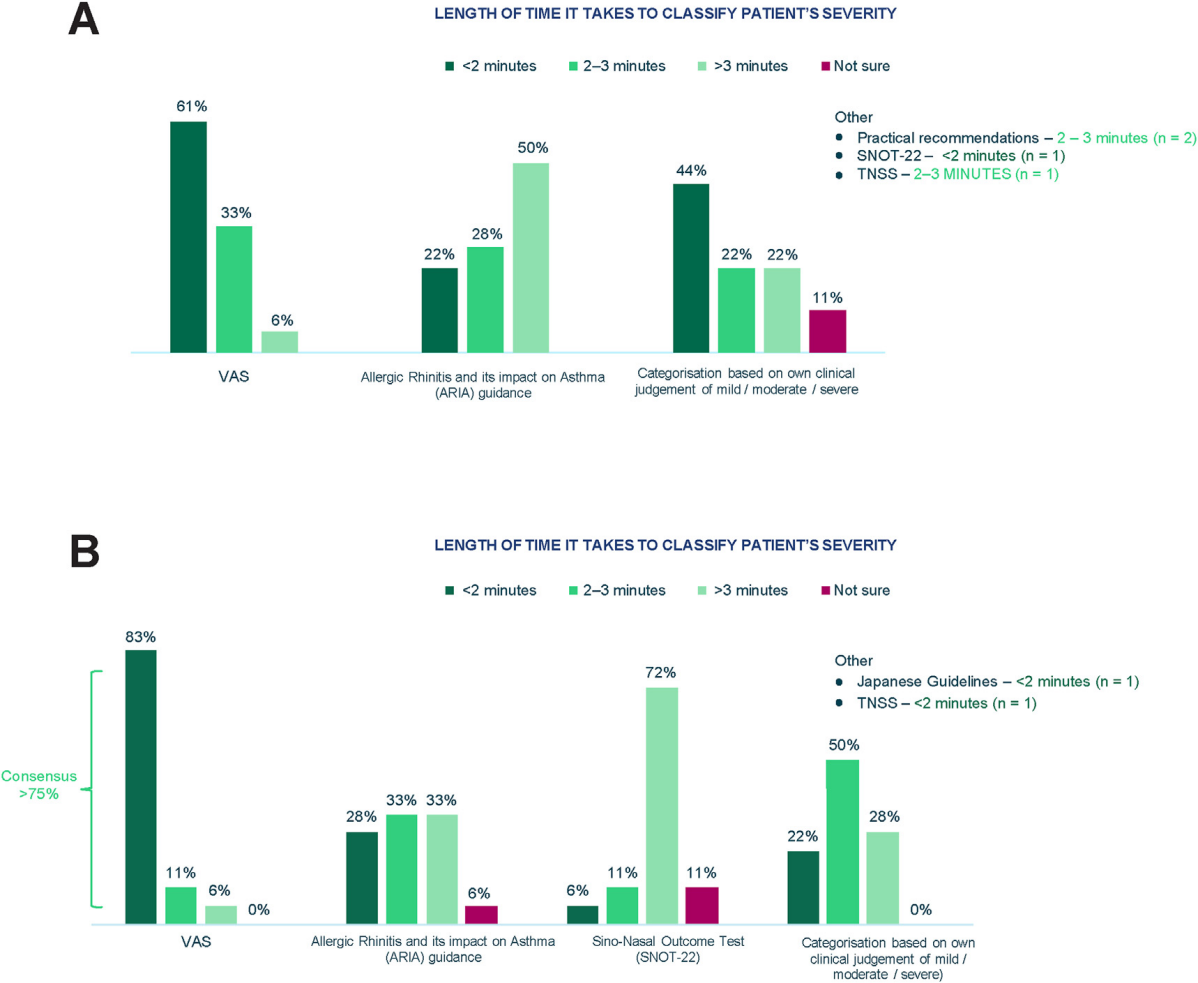
First-round (A) and second-round (B) questionnaire findings on the length of time it takes to classify patient severity. A: First-round questionnaire, Q7: How long does it take to classify a patient's severity using these scales? Base: N = 18. B: Second-round questionnaire, Q3: How long does it take to classify a single patient's severity using these scales? Base: N = 18. ARIA, Allergic Rhinitis and Its impact on Asthma; Q, question; SNOT-22, sino-nasal outcome test; TNSS, total nasal symptoms score; VAS, visual analogue scale

While consensus was not reached in the first-round questionnaire on the time it takes to classify a patient's severity using the VAS, ARIA, and clinical judgment (Fig. 3A), when respondents were asked specifically about classifying an individual patient, there was consensus that it takes less than 2 min to classify a single patient's severity using the VAS (15/18 respondents) (Fig. 3B).

In addition to the VAS and ARIA guidelines, the sino-nasal outcome test (SNOT-22),^17^ Japanese guidelines,^13^ and total nasal symptoms score (TNSS)^16^ were mentioned as alternative tools used to determine severity and assess symptom control in patients with AR.

### Global opinions vary on treatment preference for AR

**Fig. 4:**
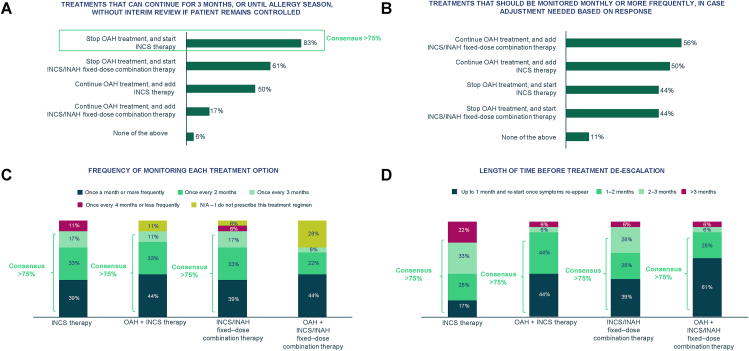
First-round (A, B) and second-round (C, D) questionnaire findings on treatment options, treatment monitoring and treatment de-escalation preferences for AR. A: First-round questionnaire, Q14: Which of the below treatment regimens, once prescribed, can continue for 3 months or until allergy season, without interim review or adjustment if the patient remains controlled? Base: N = 18. B: First-round questionnaire, Q15: Which of the above treatment regimens, once prescribed, should be monitored monthly or more frequently, in case treatment adjustment is required, based on response to treatment (including no response)? Base: N = 18. C: Second-round questionnaire, Q6: In practice, how frequently do you monitor patients on each of the following treatments, in case treatment adjustment is required based on response to treatment (including no response)? Base: N = 18. Second-round questionnaire, Q7: Q7: For how long do you recommend keeping a patient on a treatment regimen, once their symptoms are controlled, before you try to de-escalate their treatment? Please indicate a timescale for each of the following treatment regimens. Base: N = 18. AR, allergic rhinitis; INAH, intranasal antihistamine; INCS, intranasal corticosteroid; N/A, not applicable; OAH, oral antihistamine; Q, question

There was consensus that, of a variety of options, stopping oral antihistamine (OAH) therapy and starting intranasal corticosteroids (INCS) for 3 months without interim review if the patient's AR remained controlled was a suitable treatment decision (15/18 respondents) (Fig. 4A). Otherwise, for most other given treatment options, responses differed on what respondents considered to be most suitable in their own opinion for the scenarios presented in the questionnaire (Fig. 4A and B), indicating personal and regional differences in daily practice.

**Fig. 5:**
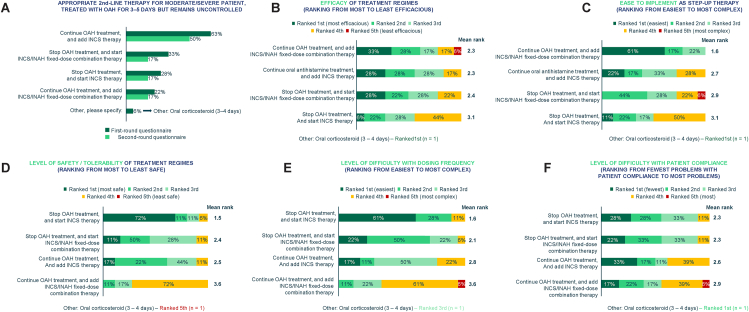
First-round (A–F) and second-round (A) questionnaire findings for treatment preferences for AR based on individual factors. A: Respondents could select multiple options. First-round questionnaire; Q8: For patients with moderate-severe allergic rhinitis, who are eligible for intranasal corticosteroid but so far have only been treated with oral antihistamine for 3–5 days and remain uncontrolled, which of the below treatment approaches would you consider to be the most appropriate 2nd-line therapy? Base: N = 18; Second-round questionnaire, Q4: For patients with moderate-severe allergic rhinitis, who are eligible for intranasal corticosteroid but so far have only been treated with OAH for at least 3–5 days and remain uncontrolled, which of the below treatment approaches would you consider appropriate as 2nd-line therapy? Base: N = 18. B: First-round questionnaire, Q10: Please rank the below treatment options based on their efficacy for patients with moderate-severe allergic rhinitis (1 = most efficacious, 4 = least efficacious)? Base: N = 18. C: First-round questionnaire, Q9: Please rank the below treatment options based on their ease to implement as step-up therapy for patients with moderate-severe allergic rhinitis (1 = easiest, 4 = most complex)? Base: N = 18. D: First-round questionnaire, Q13: Please rank the below treatment options based on their safety/tolerability for patients with moderate-severe allergic rhinitis (1 = most safe, 4 = least safe)? Base: N = 18. E: First-round questionnaire, Q11: Please rank the below treatment options based on their level of dosing frequency difficulty (1 = easiest dosing frequency, 4 = most complex dosing frequency)? Base: N = 18. F: First-round questionnaire, Q12: Please rank the below treatment options based on their difficulty level in terms of patient compliance (1 = fewest problems with patient compliance, 4 = most problems with patient compliance)? Base: N = 18. AR, allergic rhinitis, INAH, intranasal antihistamine; INCS, intranasal corticosteroid; OAH, oral antihistamine; OCS, oral corticosteroid

Also, when presented with a variety of second-line therapy options for a patient with moderate–severe AR with uncontrolled symptoms after receiving OAH for 5 days, consensus was lacking among respondents when asked to identify appropriate treatments (Fig. 5A), and to rank treatment options based on several factors (efficacy, easiest to implement as step-up therapy, safety/tolerability, ease of dosing frequency, and patient compliance) (Fig. 5B–F). This remained the case when individual factors were combined into a single question whereby there was agreement on starting INCS as a preferred therapy in this scenario; however, there was no consensus on whether OAH therapy should be continued or not when starting INCS therapy (Fig. 5A) – although two-thirds of respondents reported that they would continue OAH therapy when adding a second-line therapy involving INCSs (OAH + INCS: 9/18 respondents; OAH + INCS/intranasal antihistamine (INAH) fixed-dose combination therapy: 3/18). Overall, there was no consensus on the most appropriate/favourable second-line therapy for patients who remained uncontrolled on OAH, and opinion was split across the treatment options for a range of factors (efficacy, ease of implementation as step-up therapy, safety/tolerability, dosing frequency, and patient compliance).

After first-line OAH therapy failure, there was not much difference in preference for second-line therapy in terms of efficacy (Fig. 5B). However, there was a larger difference in preference when respondents were asked to consider the safety of the treatment options listed (Fig. 5D). In general, most respondents (16/18) considered INCS therapy with/without OAH as the safest second-line therapy option, and OAH with INCS/INAH fixed-dose combination therapy was reported as the most effective therapy (Fig. 5B), despite being deemed the most concerning in terms of safety (Fig. 5D) and the most complicated in terms of dosing regimen (Fig. 5E). Respondents considered INCS with/without OAH to have the easiest dosing frequency. Overall, INCS monotherapy was the most popular treatment option selected by respondents (Fig. 5D–F).

Open response questions on rationale for treatment preference found the following:

• Combination therapy was seen as the most efficacious way to treat AR due to the cumulative effect of the treatments
• Respondents believed it was easier to add a treatment to a regimen, rather than switching to a different treatment
• Monotherapy INCS was regarded as the safest treatment option as there were some concerns over adverse events when combining OAH and INCS
• Stopping OAH and starting INCS treatment was regarded as the easiest regimen in terms of dosing frequency
• Dosing regimens were thought to be easier when there was a single medication for patients to take
• In general, respondents shared that they felt the best options for patient compliance were the ones that have a mix of good efficacy and easy-to-follow dosing.

One respondent suggested using oral corticosteroid therapy for 3–4 days as a second-line therapy, and for multiple treatment preference questions, ranked it above the 4 options listed within the original survey (OAH, OAH + INCS, OAH + INCS/INAH fixed-dose combination therapy and INCS/INAH fixed-dose combination therapy only). No other treatments, such as leukotriene receptor antagonists, were suggested by the questionnaire respondents.

The only other areas of consensus in terms of treatment approach were the responses on how frequently treatments should be monitored, with 16/18 respondents agreeing INCS, OAH + INCS or INCS/INAH fixed-dose combination therapies could be monitored every 1–3 months (Fig. 4C), and the length of time to allow before considering stepping down a treatment (for each presented treatment option, at least 14/18 respondents agreed treatment could be de-escalated after 1–3 months of controlled AR symptoms) (Fig. 4D).

Almost two-thirds of respondents (11/18) agreed they would use allergen immunotherapy (AIT) in at least some of their patients, with 4/18 respondents reporting they would administer AIT in all cases (full details in the Supplemental Appendix).

When asked about the use of as-needed/*pro re nata* (PRN) treatment in moderate–severe AR, more than half of the respondents (10/18) said they did recommend PRN treatment (Yes, frequently: 3/18 respondents; Yes, occasionally: 7/18 respondents; No, never: 8/18 respondents). Of those who answered yes, 7/10 respondents said they would feel comfortable prescribing OAH on a PRN basis, with 7/10 respondents providing this confirmation for INCS therapy and 5/10 respondents opting for INCS/INAH fixed-dose combination therapy, citing seasonal AR as the main reason for recommending the treatment.

When asked about their motivations for recommending surgery, 8/18 respondents would only recommend surgery if the patients’ symptoms remained uncontrolled on third-line therapy, and some would recommend surgery due to certain anatomical abnormalities such as nasal blockages and significant septal deviation.

## Discussion

Our study, the first modified Delphi panel study to focus solely on AR, highlights a certain degree of consensus among AR experts from different global regions in the diagnosis, classification, and treatment of AR. While fewer questions were asked about AR diagnosis versus AR classification and treatment, these responses elicited the largest agreement among the panellists. There was consensus on the factors that should contribute most to an AR diagnosis, but respondents did not have a definitive preference for a particular tool (validated or unvalidated) to use in classifying a patient's severity during consultations.

Most strikingly, our study findings show how far daily practice can differ from guideline recommendations in some global regions. Thus, even though guideline recommendations are clear and well-supported by evidence, there was little consensus on how best to treat patients with AR in the scenarios presented to participants. This divergence of opinion may in part reflect the role that patient access factors and differences between healthcare reimbursement systems have on the availability of different medications for treating AR within and between different countries/regions.^24^ For example, during the panel discussion, some respondents remarked that INCS/INAH is not available or can only be prescribed at certain level of healthcare in some regions.

Respondents generally concluded that INCS therapy was a safe treatment option, with or without OAH therapy. As INCS therapy can take up to 2 weeks to have the full therapeutic effect,^25^ initial second-line combination therapy with OAH may be preferred; thereafter, and when AR symptoms are controlled, the OAH can be discontinued, leaving the patient on INCS monotherapy.

Regarding efficacy, respondents had a similar preference among the different treatment options as second-line therapy after OAH failure, with OAH plus INCS/INAH fixed-dose combination therapy marginally favoured. In general, respondents reported that continuing OAH therapy and adding supplementary treatment was considered more efficacious than stopping OAH and switching therapy. However, this treatment option was ranked lowest for safety, ease-of-dosing and patient compliance, highlighting the need to consider a variety of treatment and patient factors when selecting a treatment course. This treatment preference is not in line with AR guidelines, which advise adding OAH to an INCS is not superior to INCS monotherapy. However, a closer inspection of the specific clinical question in the Practice Parameter on Rhinitis 2020 Update^3^ reveals that this analysis was for initial treatment of AR and there are few trials designed to investigate second-line therapy options. As such, the alternative treatment options after first-line treatment failure are purely advisory in these guidelines, due to a lack of evidence.

Considerable panel discussion around the stepping up/down of treatment led to the inclusion of questions in the second-round questionnaire on how often patients receiving particular therapies should be monitored, and the length of time before a physician may consider stepping down a treatment. For both questions, respondents reached a consensus for all 4 treatment options (except monitoring of OAH plus INCS/INAH fixed-dose combination therapy; 72%); however, there was some disparity of opinion regarding the specific time period (≤1 month, 1–2 months, 2–3 months) before stepping down the different treatment options. This is thought to be related to the type of AR predominant in a given population: for example, in regions where seasonal AR is most common, physicians may de-escalate treatment more rapidly out-of-season, while physicians working in more tropical regions (eg, Brazil and Mexico), where house dust mites are the primary allergen responsible for perennial AR cases,^14^^,^^26^^,^^27^ may opt for continuing therapy for at least 2–3 months before de-escalating therapy. Generally, step down of treatment is considered in patients with mild or controlled symptoms; for those with moderate–severe AR who have not improved after 2 weeks of INCS therapy, stepping up treatment by referral to a specialist and the use of other therapies, such as allergen immunotherapy or surgery, are considered.^1^

There was interest among physicians in exploring PRN dosing, specifically in patients with milder, intermittent symptoms and those with seasonal AR who experienced exacerbations out-of-season. Opinion on the use of PRN therapy indicated that, for more than half of the respondents considered for intermittent, mild or out-of-season patients, prescribing PRN therapy could be appropriate, as specified by panellists during the expert panel discussion; however, when asked for PRN prescribing in the moderate–severe patient case presented in the second-round questionnaire, there was a lack of consensus on the appropriateness of this treatment strategy, considered too mild by some respondents. Multiple respondents commented during the expert panel discussion that once a patient's symptoms become controlled, the patient may independently start using their prescribed therapy on an as-needed basis. For PRN prescribing, OAH and INCS were the most popular treatment options among respondents; however, it should be noted that PRN is an off-label indication for INCS.

The promise of PRN therapy in managing AR is supported by recent updates to AR guidelines^1^ and recent clinical studies.^19^^,^^28^, ^29^, ^30^, ^31^, ^32^ Additional descriptive analyses have also been carried out.^31^ Two analyses by the Mobile Airways Sentinel Network (MASK)^28^, ^29^, ^30^ were designed to utilise data from more than 9000 users from 22 countries, confirming results from a pilot study.^28^ Regarding PRN use, the studies reported that patients were poorly adherent to treatment,^28^^,^^30^^,^^31^ that no treatment trajectory could be identified,^29^ and that most patients self-medicated. In the MASK study, most patients with AR used on-demand treatment when their symptoms were sub-optimally controlled. When symptoms were uncontrolled, patients changed their medications daily for control and typically started to use 3–5 different medications.^28^ Furthermore, the MASK dataset demonstrated that the vast majority of patients did not follow guidelines or physicians' prescriptions.^28^, ^29^, ^30^ Additionally, the MASK studies found physicians behaved like patients^33^ when they themselves were experiencing allergy-related symptoms, suggesting the need for behavioural science to promote improved control. Furthermore, patients who did not take medications usually had well-controlled symptoms,^28^^,^^29^ while increasing medication use was related to worse AR control in most patients.^28^^,^^29^ These results indicated that, when a patient's symptoms were controlled, they either did not take medication or remained on a single treatment, and when a patient's symptoms were uncontrolled, they co-medicated using multiple therapies;^1^^,^^31^ these treatment patterns were found to be similar across European regions.^31^ The results in the MASK studies also showed that co-medication for AR, such as OAH + INCS, did not have a measurable benefit.^1^ A recent systematic review was designed to assess the effectiveness of PRN INCS for treating AR.^32^ Eight studies involving a total of 882 participants met the criteria.^19^^,^^32^^,^^34^, ^35^, ^36^, ^37^, ^38^, ^39^, ^40^ While regular INCS use improved TNSS and disease-specific quality of life more than PRN INCS, PRN INCS improved TNSS more than PRN antihistamine and placebo.^32^

Panel responses were inconclusive when they were asked about when surgery for AR should be considered. Patient stratification for surgery was generally considered to include anatomical anomalies such as: concha bullosa; obstructive septal deviation; other comorbidities such as nasal polyps, inferior turbinates enlargement and chronic rhinosinusitis; and poor control of symptoms, despite appropriate pharmacotherapy according to guidelines. It should be noted, however, that although surgery may not treat the mechanisms underlying AR,^1^ it could be useful to improve symptoms. Patients need sufficient explanation for the merits, potential complications and adverse events of turbinate-reducing surgery.

Although there are disparities between real-world practice and guideline recommendations, several groups of guideline developers^2^^,^^9^^,^^14^ are working to narrow knowledge gaps through educational initiatives with physicians (eg, online webinars and off-line on-demand courses) and patients (eg, the MASK-air diary as part of the aforementioned MASK study). Additionally, the ARIA severity classification which was promoted in 2008 (mild–moderate–severe AR) was recently replaced by the VAS scale.^2^^,^^18^

While there are no other published modified Delphi panel studies focussed solely on AR, there have been recent studies designed to establish consensus among healthcare practitioners on the differences in the signs and symptoms of COVID-19 versus AR and the common cold,^41^ the use of biologic therapies for chronic rhinosinusitis in Canada^42^ and in the rhinology-specific priority setting in the United States.^43^ Other Delphi panel studies have been published on more general respiratory medicine topics such as airway management,^44^ achieving asthma remission as a treatment goal,^45^ or to inform AR guideline development^46^; however, these studies include different methodological approaches to that used herein, such as the inclusion of accompanying literature reviews. Our study adds to this body of work.

Our study has several strengths. A notable strength of this modified Delphi panel study is that individual respondents can share their opinion anonymously without being influenced by the wider panel. Participants in our study were recruited using strict criteria; thus, their responses and opinions reflect current knowledge and perceptions. Additionally, the second questionnaire was able to resolve some areas in which consensus was lacking from the first questionnaire, confirming the goal of our modified Delphi panel study design.^23^

In terms of limitations, we acknowledge that 18 respondents may appear to be a relatively small sample size, but it is in accordance with recommendations for modified Delphi panel studies and is within the optimal range to ensure accurate interpretation of findings, as too large a group can lead to reduced degree of generalisability.^20^^,^^22^ Even though we are able to include details on the different respondents’ specialties, we note that we were unable to stratify the respondents according to their specialty. Additionally, as there is no universally accepted definition of the minimum percentage required to reach a consensus in a Delphi panel study, thus the >75% threshold used in our study could be adjusted, and the survey results reinterpreted. Finally, in our study we specifically focused on the pharmacological management of AR. As such, we did not include other aspects in treating a patient with AR, such as shared decision making, nasal washes, allergen avoidance, and electronic monitoring.

## Conclusions

Our two-part modified Delphi panel study identified some areas of consensus among AR experts in terms of diagnosis, classification and treatment of AR. Regarding diagnosis, physicians use a combination of tests during a patient consultation and tend to collaborate with colleagues across other specialties (eg, ENT specialists with allergists) as part of their diagnosis efforts. When classifying AR, respondents agreed on considering severity, involvement of the upper/lower respiratory tract and the frequency of symptoms as the most important factors; however, there was no decisive preference for which classification tool is best used when given a choice between the VAS and ARIA guidelines. In terms of treating AR, physicians were largely in agreement that INCS monotherapy required less monitoring versus other given treatment options, and they also agreed on when treatments should be stepped down. There were mixed opinions, however, on the best course of treatment in the presented case studies, and on when PRN and surgery should be considered for patients with moderate–severe AR. Overall, our findings are useful for better understanding of current global clinical practices in the diagnosis, classification and treatment of AR, and indicate that clear differences exist across local practice.

## Abbreviations

AR, allergic rhinitis; ARIA, Allergic Rhinitis and its Impact on Asthma; ENT, ear, nose and throat; IgE, immunoglobulin E; INAH, intranasal antihistamine; INCS, intranasal corticosteroid therapies; MASK, Mobile Airways Sentinel Network; N/A, not applicable; OAH, oral antihistamine; PRN, as-needed/*pro re nata* treatment; Q, question; RAST, radio-allergosorbent test serum specific IgE test; SNOT-22, sino-nasal outcome test; TNSS, total nasal symptoms score; VAS, visual analogue scale.

## Acknowledgments

The authors wish to thank Prof. Oxana Kurbacheva, Prof. Tatiana Fedoskova, Prof. Andrey Lopatin, Dr Guillermo Plaza Mayor, Asst. Prof. Sira Nanthapisal, Prof. Carlos de la Torre, Prof. Natalia Nenasheva, Dr Rosa Stolle Arranz, Dr Isam Alobid and Dr Juan Carlos Fernandes de Cordova for contributing to the two questionnaires. Medical writing support, under the guidance of the authors, was provided by Fiona Scott, PhD, of Ashfield MedComms (Glasgow, UK), an Inizio company, and was funded by GSK.

## Financial support and conflict of interest disclosure

This study, including study design, data collection, analysis, and interpretation, and medical writing and submission support for the manuscript, was funded by GSK (study 114971). Delphi questionnaire panellists were recruited directly by GSK, were aware that the study was being funded by GSK and did not receive any honoraria for their participation. Medical writing support provided by Fiona Scott, PhD, Ashfield MedComms (Glasgow, UK), an Inizio company.

**DESLL** reports the following outside of the submitted work: speaker, advisory board and safety board member for ALK, Allakos, Amstrong, 10.13039/100004325AstraZeneca, Carnot, 10.13039/100019719Chiesi, 10.13039/501100011831DBV Technologies, Laboratorios Grin, Grunenthal, GSK, Liomont, Viatris, Menarini, 10.13039/100009947Merck Sharp & Dohme (MSD), 10.13039/100004336Novartis, 10.13039/100004319Pfizer, 10.13039/100004339Sanofi, Siegfried, and UCB; and travel and educational grants from 10.13039/100006483Abbvie, 10.13039/100004325AstraZeneca, 10.13039/100004326Bayer, Circassia, GSK, Lilly, Novartis, 10.13039/100004319Pfizer, Purina Institute, 10.13039/100004339Sanofi, and 10.13039/100011110UCB.

**JLMB** reports the following outside of the submitted work: grants from 10.13039/100004326Bayer, Carnot, 10.13039/100019719Chiesi, Glenmark, Gruenthal, MSD, and Siegfried.

**JMS** reports the following outside of the submitted work: grants from Amplifon (GAES), 10.13039/100004325AstraZeneca, Viatris, 10.13039/100004336Novartis, and 10.13039/100004339Sanofi.

**AVE** reports the following outside of the submitted work: payment or honoraria for lectures, presentations, speakers bureaus, manuscript writing or educational events from ALK, AstraZeneca, Berlin Chemie, Chiesi, GSK, Novartis, Sandoz, Sanofi, and Stallergenes.

**RLLD** reports the following outside of the submitted work: payment for lecture from Sanofi and advisory board member for GSK.

**MMM** reports the following outside of the submitted work: grants and consulting fees from Myralis Pharma; payment or honoraria for lectures, presentations, speakers bureaus, manuscript writing, or educational events from Myralis Pharma and GSK; and support for attending meetings and/or travel from 10.13039/100004339Sanofi.

**YO** reports the following outside of the submitted work: grants from Allergologisk Laboratorium København, Kirin, Mitsubishi-Tanabe, 10.13039/100004336Novartis and Torii Co ltd.

## Availability of data and materials

The authors confirm that the data supporting the findings of this study are available within the article and supplementary information.

## Agreement to publish the work

All authors agree to the publication of this work in the *World Allergy Organization Journal*.

## Statement of contribution to the work

DESLL, JLMB, JMS, AVE, RLLD, MMM, and YO had access to the study data, take responsibility for the accuracy of the analysis, contributed to data interpretation, reviewed, and contributed to the content of the manuscript, and had authority in the decision to submit the manuscript.

## Ethics statement

This study complied with all applicable laws regarding participant privacy. No direct participant contact, or primary collection of individual human subject data occurred. Study results were in tabular form and aggregate analyses omitted participant identification, therefore informed consent, ethics committee, or Institutional Review Board approval were not required.

## Editorial policy confirmation and agreement

All authors confirm they agree to *World Allergy Organization's* editorial policies.

## Confirmation of unpublished work

The authors confirm the following: their manuscript is original and has not been published in full before; is not currently being considered for publication elsewhere; and has not been posted to a preprint server. Some of the material discussed in this manuscript was previously presented at the European Academy of Allergy and Clinical Immunology (EAACI) Hybrid Congress 2022, Prague, Czech Republic, and Virtual, 1–3 July 2022; Larenas-Linnemann et al., “Expert Consensus Using the Modified Delphi Technique on Tools for Allergic Rhinitis Severity Classification and Treatment Options in Day-to-Day Clinical Practice”.
