# Supplementary material for: Global expert views on the diagnosis, classification and pharmacotherapy of allergic rhinitis in clinical practice using a modified Delphi panel technique
Source: World Allergy Organ J. 2023 Jul 17;16(7):100800. doi: 10.1016/j.waojou.2023.100800 (PMC10372170; doi:10.1016/j.waojou.2023.100800)
Supplement: Multimedia component 1 [file mmc1.pdf]

Global Expert Views on Diagnosing, Classifying and Treating Allergic Rhinitis in Clinical Practice: A Modified Delphi Method Questionnaire Study

Supplemental Appendix

Supplementary Figure 1 Development of second-round questionnaire on AR diagnosis and classification from first-round questionnaire

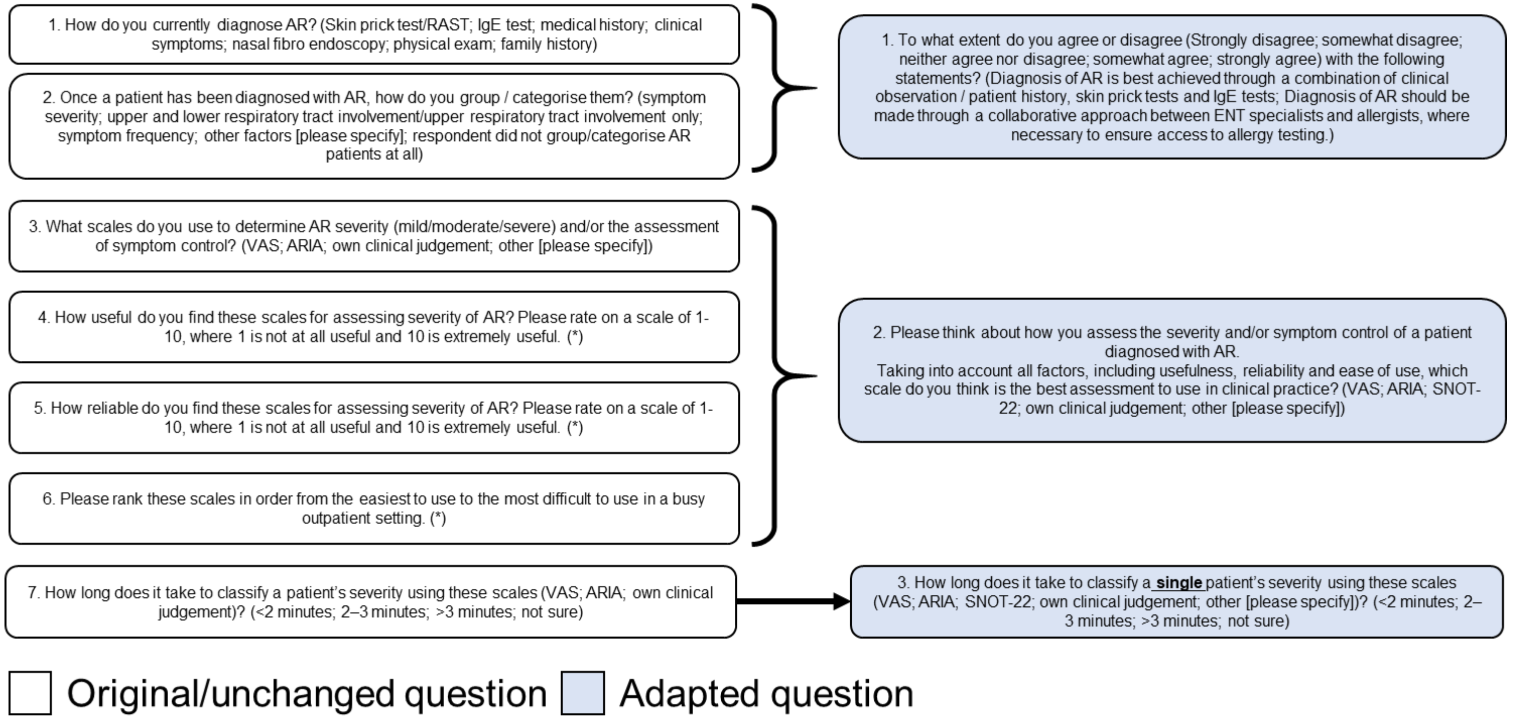

*Response options: VAS; ARIA; own clinical judgement. AR, allergic rhinitis; ARIA, Allergic Rhinitis and its Impact on Asthma; ENT, ear, nose and throat; IgE, immunoglobulin E; RAST, radio-allergosorbent (serum specific IgE) test; SNOT-22, sino-nasal outcome test; VAS, visual analogue scale.

Supplementary Figure 2 Development of second-round questionnaire questions on AR treatment from first-round questionnaire

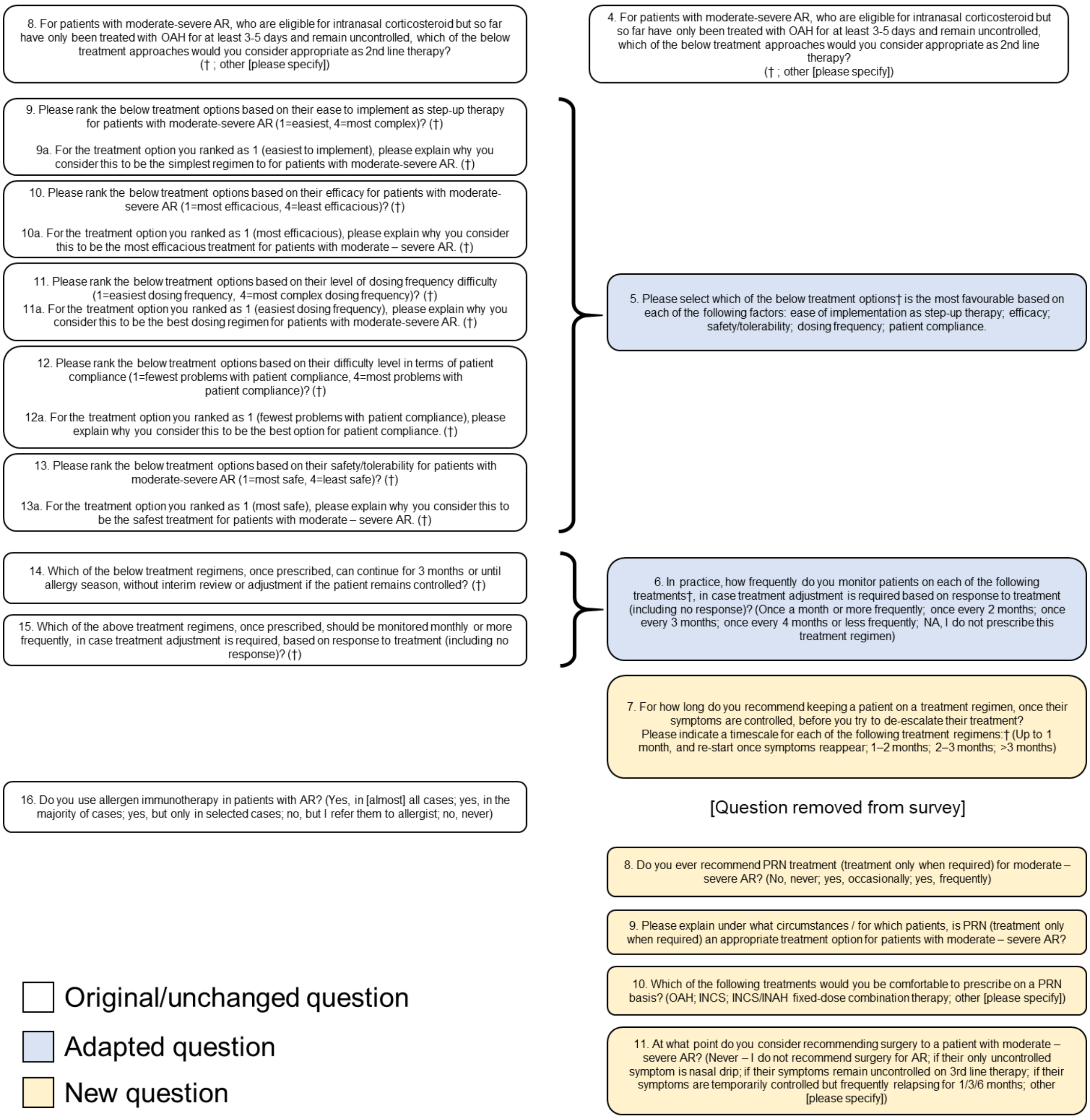

†Response options: Continue OAH therapy and add INCS therapy; continue OAH therapy and add INCS/INAH fixed dose combination therapy; stop OAH therapy and start INCS therapy; stop OAH therapy and start INCS/INAH fixed-dose combination therapy.

AR, allergic rhinitis; INAH, intranasal antihistamine; INCS, inhaled corticosteroid; OAH, oral antihistamine; PRN, as needed.

Full Delphi questionnaires *First-round questionnaire*

Q	Question	Answers	Programming notes (internal use)
	**Screening**		
INTRO	Thank you for agreeing to take part in GSK's expert panel on the topic of allergic rhinitis.<br>In preparation for the initial workshop, we ask you to complete the following questionnaire.<br><br>The purpose of this questionnaire is to understand the diagnostic tools and therapeutic approaches physicians are using to diagnose and treat their patients with uncontrolled moderate–severe allergic rhinitis.<br><br>It should take approximately 10 minutes and should be completed by [Date].		

	The questionnaire is for information gathering purposes only and it is not intended to be promotional. The questionnaire is being conducted by Ashfield MedComms on behalf of GSK.<br><br>This information-gathering exercise complies with UK Data Protection law (GDPR) and with the British Healthcare Business Intelligence Association's (BHBIA) Legal & Ethical Guidelines, along with the European Pharmaceutical Market Research Association's (EphMRA).<br><br>Any information you provide us with will be kept **confidential and anonymous** and will be combined with feedback from the other attendees at the workshop.<br><br>Your information will only be used for this expert panel and will not be passed to any other organisation without your permission. Your participation is voluntary, and you have the right to withdraw at any time.<br><br>For more information about your rights please see our privacy notice: https://www.evokegroup.com/privacy-notice-for-market-research-and-survey-participants	

	**Adverse events**<br><br>We are required to pass on to GSK, a pharmaceutical company, details of adverse events/product complaints related to their own product(s) that are mentioned during the course of this questionnaire. Although how you respond will, of course, be treated in confidence, should you raise an adverse event/product complaint in a specific patient or group of patients, we will need to report this, even if it has already been reported by you directly to the company or the regulatory authorities. If you decide to disclose your personal details in association with any adverse event/product complaint report, this information will be disclosed to the commissioning company. In such a situation you may be contacted specifically in relation to that adverse event/product complaint. Everything else you contribute during the course of the interview will continue to remain confidential.		
AE	Are you happy to proceed with the questionnaire on this basis?	1. Yes<br>2. No	SINGLE SELECT<br>CLOSE IF NO

	**Questionnaire**		
	Great, let's begin!<br><br>We are interested to understand about your prescribing preferences for the treatment and management of patients with allergic rhinitis.		
Q1	How do you currently diagnose allergic rhinitis?<br><br>Please explain fully.		OPEN RESPONSE
Q2	Once a patient has been diagnosed with allergic rhinitis, how do you group / categorise them?	1. I group them based on level of severity<br>2. I group them based on frequency of symptoms<br>3. I group them based on whether there is involvement of both the upper & lower respiratory tract, or only the upper respiratory tract	MULTI SELECT

		4. I group them based on other factors. Please specify________ [OPEN]<br>5. I do not group / categorise allergic rhinitis patients at all [EXCLUSIVE]	
Q3	What scales do you use to determine allergic rhinitis severity (mild / moderate / severe) and / or the assessment of symptom control?	1. Visual Analogue Scale (VAS)<br>2. Allergic Rhinitis and its impact on Asthma (ARIA) guidance<br>3. I categorise them based on my own clinical judgement of mild / moderate / severe<br>4. Other, please specify_____[OPEN]<br>5. Other, please specify_____[OPEN]<br>6. Other, please specify_____[OPEN]	MULTI SELECT<br>ONLY SHOW IF SELECT (1) AT Q2
Q4	**How useful** do you find these scales for assessing **severity of allergic rhinitis**?	Rows:<br>1. Visual Analogue Scale (VAS)	GRID RATING SCALE

	Please rate on a scale of 1-10, where 1 is not at all useful and 10 is extremely useful.	2. Allergic Rhinitis and its impact on Asthma (ARIA) guidance<br>3. Categorisation based on own clinical judgement of mild / moderate / severe<br>4. Other, please specify_____[OPEN]<br>5. Other, please specify_____[OPEN]<br>6. Other, please specify_____[OPEN]<br><br>Columns:<br>1 (not at all useful) – 10 (extremely useful)	PIPE THROUGH ANY OTHER OPTIONS ADDED AT Q3<br><br>INCLUDE POP UP DEFINITIONS OF VAS AND ARIA<br><br>VAS: Psychometric measuring instruments designed to document symptom severity and disease control

			in individual patients.<br><br>ARIA: Guidelines classifying allergic rhinitis as “intermittent” or “persistent”, depending on the presence of symptoms within 4 weeks.
Q5	**How reliable** do you find these scales for assessing **severity of allergic rhinitis**? Please rate on a scale of 1-10, where 1 is not at all reliable and 10 is extremely reliable.	Rows:<br>1. Visual Analogue Scale (VAS)<br>2. Allergic Rhinitis and its impact on Asthma (ARIA) guidance	GRID RATING SCALE<br>PIPE THROUGH ANY OTHER

		3. Categorisation based on own clinical judgement of mild / moderate / severe<br>4. Other, please specify_____[OPEN]<br>5. Other, please specify_____[OPEN]<br>6. Other, please specify_____[OPEN]<br><br>Columns:<br>1 (not at all reliable) – 10 (extremely reliable)	OPTIONS ADDED AT Q3<br><br>INCLUDE POP UP DEFINITIONS OF VAS AND ARIA
Q6	Please rank these scales in order from the easiest to use to the most difficult to use in a **busy outpatient setting.**	1. Visual Analogue Scale (VAS)<br>2. Allergic Rhinitis and its impact on Asthma (ARIA) guidance<br>3. Categorisation based on own clinical judgement of mild / moderate / severe<br>4. Other, please specify_____[OPEN]<br>5. Other, please specify_____[OPEN]<br>6. Other, please specify_____[OPEN]	RANKING<br>PIPE THROUGH ANY OTHER OPTIONS ADDED AT Q3

			INCLUDE POP UP DEFINITIONS OF VAS AND ARIA
Q7	How long does it take to classify a patient's severity using these scales?	Rows:<br>1. Visual Analogue Scale (VAS)<br>2. Allergic Rhinitis and its impact on Asthma (ARIA) guidance<br>3. Categorisation based on own clinical judgement of mild / moderate / severe<br>4. Other, please specify_____[OPEN]<br>5. Other, please specify_____[OPEN]<br>6. Other, please specify_____[OPEN]<br><br>Columns:<br>1. <2 minutes<br>2. 2-3 minutes	GRID RATING SCALE<br>PIPE THROUGH ANY OTHER OPTIONS ADDED AT Q3<br><br>INCLUDE POP UP DEFINITIONS OF VAS AND ARIA

		3. >3 minutes<br>4. Not sure	
Q8	For patients with **moderate-severe allergic rhinitis,** who are **eligible for intranasal corticosteroid** but so far have **only been treated with OAH for at least 3-5 days and remain uncontrolled**, which of the below treatment approaches would you consider **appropriate** as 1st line therapy?<br>*Please select all that apply*	1. Stop oral antihistamine treatment, and start intranasal corticosteroid therapy<br>2. Continue oral antihistamine treatment, and add intranasal corticosteroid therapy<br>3. Stop oral antihistamine treatment, and start intranasal corticosteroid/Intranasal antihistamine fixed-dose combination therapy<br>4. Continue oral antihistamine treatment, and add intranasal corticosteroid/intranasal antihistamine fixed-dose combination therapy<br>5. Other, please specify____[OPEN]	MULTI SELECT

Q9	Please rank the below treatment options based on their **ease to implement** as step-up therapy for patients with moderate-severe allergic rhinitis (1=easiest, 4=most complex)?	1. Stop oral antihistamine treatment, and start intranasal corticosteroid therapy<br>2. Continue oral antihistamine treatment, and add intranasal corticosteroid therapy<br>3. Stop oral antihistamine treatment, and start intranasal corticosteroid/intranasal antihistamine fixed-dose combination therapy<br>4. Continue oral antihistamine treatment, and add intranasal corticosteroid/intranasal antihistamine fixed-dose combination therapy	RANK ORDER 1-4<br>PIPE THROUGH 'OTHER' RESPONSE FROM Q8 IF SELECTED, AS A 5TH OPTION

Q9a	For the treatment option you ranked as 1 (easiest to implement), please explain why you consider this to be the simplest regimen to implement for patients with moderate-severe allergic rhinitis.		OPEN RESPONSE
Q10	Please rank the below treatment options based on their **efficacy** for patients with moderate-severe allergic rhinitis (1=most efficacious, 4=least efficacious)?	1. Stop oral antihistamine treatment, and start intranasal corticosteroid therapy<br>2. Continue oral antihistamine treatment, and add intranasal corticosteroid therapy<br>3. Stop oral antihistamine treatment, and start intranasal corticosteroid/intranasal antihistamine fixed-dose combination therapy<br>4. Continue oral antihistamine treatment, and add intranasal	RANK ORDER 1-4 PIPE THROUGH 'OTHER' RESPONSE FROM Q8 IF SELECTED, AS A 5TH OPTION

		corticosteroid/intranasal antihistamine fixed-dose combination therapy	
Q10a	For the treatment option you ranked as 1 (most efficacious), please explain why you consider this to be the most efficacious treatment for patients with moderate – severe allergic rhinitis.		OPEN RESPONSE
Q11	Please rank the below treatment options based on their **level of dosing frequency difficulty** (1=easiest dosing frequency, 4=most complex dosing frequency)?	1. Stop oral antihistamine treatment, and start intranasal corticosteroid therapy<br>2. Continue oral antihistamine treatment, and add intranasal corticosteroid therapy<br>3. Stop oral antihistamine treatment, and start intranasal corticosteroid/intranasal antihistamine fixed-dose combination therapy	RANK ORDER 1-4 PIPE THROUGH ‘OTHER’ RESPONSE FROM Q8 IF SELECTED, AS A 5TH OPTION

		4. Continue oral antihistamine treatment, and add intranasal corticosteroid/intranasal antihistamine fixed-dose combination therapy	
Q11a	For the treatment option you ranked as 1 (easiest dosing frequency), please explain why you consider this to be the best dosing regimen for patients with moderate-severe allergic rhinitis.		OPEN RESPONSE
Q12	Please rank the below treatment options based on their **difficulty level in terms of patient compliance** (1=fewest problems with patient compliance, 4=most problems with patient compliance)?	1. Stop oral antihistamine treatment, and start intranasal corticosteroid therapy<br>2. Continue oral antihistamine treatment, and add intranasal corticosteroid therapy<br>3. Stop oral antihistamine treatment, and start intranasal corticosteroid/intranasal	RANK ORDER 1-4 PIPE THROUGH ‘OTHER’ RESPONSE FROM Q8 IF SELECTED, AS A 5TH OPTION

		antihistamine fixed-dose combination therapy<br>4. Continue oral antihistamine treatment, and add intranasal corticosteroid/intranasal antihistamine fixed-dose combination therapy	
Q12a	For the treatment option you ranked as 1 (fewest problems with patient compliance), please explain why you consider this to be the best option for patient compliance.		OPEN RESPONSE
Q13	Please rank the below treatment options based on their **safety/tolerability** for patients with moderate-severe allergic rhinitis (1=most safe, 4=least safe)?	1. Stop oral antihistamine treatment, and start intranasal corticosteroid therapy<br>2. Continue oral antihistamine treatment, and add intranasal corticosteroid therapy	RANK ORDER 1-4<br>PIPE THROUGH 'OTHER' RESPONSE FROM Q8 IF

		3. Stop oral antihistamine treatment, and start intranasal corticosteroid/intranasal antihistamine fixed-dose combination therapy<br>4. Continue oral antihistamine treatment, and add intranasal corticosteroid/intranasal antihistamine fixed-dose combination therapy	SELECTED, AS A 5TH OPTION
Q13a	For the treatment option you ranked as 1 (most safe), please explain why you consider this to be the safest treatment for patients with moderate – severe allergic rhinitis.		OPEN RESPONSE
Q14	Which of the below treatment regimens, once prescribed, can **continue for 3 months or until allergy season, without interim review**	1. Stop oral antihistamine treatment, and start intranasal corticosteroid therapy	MULTI RESPONSE<br>PIPE THROUGH 'OTHER'

	**or adjustment if the patient remains controlled**? *(Select all that apply)*	2. Continue oral antihistamine treatment, and add intranasal corticosteroid therapy<br>3. Stop oral antihistamine treatment, and start intranasal corticosteroid/intranasal antihistamine fixed-dose combination therapy<br>4. Continue oral antihistamine treatment, and add intranasal corticosteroid/Intranasal antihistamine fixed-dose combination therapy<br>5. None of the above [EXCLUSIVE]	RESPONSE FROM Q8 IF SELECTED, AS A 5TH OPTION
Q15	Which of the above treatment regimens, once prescribed, **should be monitored monthly or more frequently**, in case treatment adjustment is required, based on response to	1. Stop oral antihistamine treatment, and start intranasal corticosteroid therapy	MULTI SELECT PIPE THROUGH 'OTHER' RESPONSE

	treatment (including no response)? *(Select all that apply)*	2. Continue oral antihistamine treatment, and add intranasal corticosteroid therapy<br>3. Stop oral antihistamine treatment, and start intranasal corticosteroid/intranasal antihistamine fixed-dose combination therapy<br>4. Continue oral antihistamine treatment, and add intranasal corticosteroid/intranasal antihistamine fixed-dose combination therapy<br>5. None of the above [EXCLUSIVE]	FROM Q8 IF SELECTED, AS A 5TH OPTION
Q16	Do you use allergen immunotherapy in patients with AR?	1. Yes in (almost) all cases<br>2. Yes, in the majority of cases<br>3. Yes, but only in selected cases<br>4. No, but I refer them to allergist	SINGLE SELECT

		5. No, never	
CLOSE	**That is the end of the questionnaire – thank you for your responses. This research is sponsored by GSK.**<br><br>If you have any final comments, please provide these below, otherwise, click to end the questionnaire.	OPTIONAL OPEN END	

Second-round questionnaire

Q	Question	Answers	Programming notes (internal use)
	**Screening**		
INTRO	Thank you for agreeing to take part in GSK's expert panel on the topic of allergic rhinitis.		

	We appreciate you completing the first questionnaire in September/October, and we would now like you to complete a follow-up questionnaire.<br><br>The purpose of this questionnaire is to gain further insight into what you believe are the optimum ways to diagnose, categorise and treat moderate – severe allergic rhinitis.<br><br>It should take approximately 10 minutes and should be completed by [Date].<br><br>The questionnaire is for information gathering purposes only and it is not intended to be promotional. The questionnaire is being conducted by Ashfield MedComms on behalf of GSK.<br><br>This information-gathering exercise complies with UK Data Protection law (GDPR) and with the British Healthcare Business Intelligence Association's (BHBIA) Legal & Ethical Guidelines, along with the European Pharmaceutical Market Research Association's (EphMRA).	

	Any information you provide us with will be kept **confidential and anonymous** and will be combined with feedback from the other attendees at the workshop.<br><br>Your information will only be used for this expert panel and will not be passed to any other organisation without your permission. Your participation is voluntary, and you have the right to withdraw at any time.<br><br>For more information about your rights please see our privacy notice: https://www.evokegroup.com/privacy-notice-for-market-research-and-survey-participants<br><br>**Adverse events**<br><br>We are required to pass on to GSK, a pharmaceutical company, details of adverse events/product complaints related to their own product(s) that are mentioned during the course of this questionnaire. Although how you respond will, of course, be treated in confidence, should you raise an adverse event/product complaint in a specific patient or group of patients, we will	

	need to report this, even if it has already been reported by you directly to the company or the regulatory authorities. If you decide to disclose your personal details in association with any adverse event/product complaint report, this information will be disclosed to the commissioning company. In such a situation you may be contacted specifically in relation to that adverse event/product complaint. Everything else you contribute during the course of the interview will continue to remain confidential.		
AE	Are you happy to proceed with the questionnaire on this basis?	1. Yes<br>2. No	SINGLE SELECT<br>CLOSE IF NO

	**Questionnaire**	
	Great, let's begin!<br><br>We are interested to understand about your prescribing preferences for the treatment and management of patients with allergic rhinitis.	

Q1	To what extent do you agree or disagree with the following statements?	**Columns**<br>1. Strongly disagree<br>2. Somewhat disagree<br>3. Neither agree nor disagree<br>4. Somewhat agree<br>5. Strongly agree<br><br>**Rows**<br>1. Diagnosis of allergic rhinitis is best achieved through a **combination** of clinical observation / patient history, skin prick tests and IgE tests<br>2. Diagnosis of allergic rhinitis should be made through a **collaborative approach** between ENT specialists	SLIDER SCALE

		and allergists, where necessary to ensure access to allergy testing	
Q2	Please think about how you assess the severity and/or symptom control of a patient diagnosed with allergic rhinitis.<br>Taking into account all factors, including usefulness, reliability and ease of use, which scale do you think is the **best assessment to use in clinical practice**?<br>*Please select one*	1. Several Visual Analogue Scales (VAS)<br>2. Allergic Rhinitis and its impact on Asthma (ARIA) guidance<br>3. Sino-Nasal Outcome Test (SNOT-22)<br>4. Categorise based solely on own clinical judgement<br>5. Other, please specify_____[OPEN]	SINGLE SELECT<br><br>INCLUDE POP UP DEFINITIONS OF VAS, ARIA, AND SNOT-22
Q3	How long does it take to classify a single patient's severity using these scales?	**Rows:**<br>1. Visual Analogue Scale (VAS)<br>2. Allergic Rhinitis and its impact on Asthma (ARIA) guidance<br>3. Sino-Nasal Outcome Test (SNOT-22)	GRID RATING SCALE<br>PIPE THROUGH ANY OTHER OPTIONS ADDED AT Q2

		4. Categorisation based on own clinical judgement of mild / moderate / severe<br>5. Other, please specify_____[OPEN]<br><br>**Columns:**<br>1. < 2 minutes<br>2. 2-3 minutes<br>3. > 3 minutes<br>4. Not sure	INCLUDE POP UP DEFINITIONS OF VAS, ARIA, AND SNOT-22
Q4	For patients with **moderate-severe allergic rhinitis,** who are **eligible for intranasal corticosteroid** but so far have **only been treated with OAH for 3-5 days and remain uncontrolled**, which of the below treatment approaches would you consider to be the	1. Stop oral antihistamine treatment, and start intranasal corticosteroid therapy<br>2. Continue oral antihistamine treatment, and add intranasal corticosteroid therapy<br>3. Stop oral antihistamine treatment, and start intranasal corticosteroid/Intranasal	SINGLE SELECT

	**most appropriate** 2nd line therapy?<br>*Please select one*	antihistamine fixed-dose combination therapy<br>4. Continue oral antihistamine treatment, and add intranasal corticosteroid/Intranasal antihistamine fixed-dose combination therapy<br>5. Other, please specify____[OPEN]	
Q5	Please select which of the below treatment options is the **most favourable** based on each of the following factors.	**Rows:**<br>1. Ease of implementation as step-up therapy<br>2. Efficacy<br>3. Safety / Tolerability<br>4. Dosing frequency<br>5. Patient compliance	GRID<br>SINGLE SELECT<br>PER ROW

		**Columns:**<br>1. Stop oral antihistamine treatment, and start intranasal corticosteroid therapy<br>2. Continue oral antihistamine treatment, and add intranasal corticosteroid therapy<br>3. Stop oral antihistamine treatment, and start intranasal corticosteroid/intranasal antihistamine fixed-dose combination therapy<br>4. Continue oral antihistamine treatment, and add intranasal corticosteroid/intranasal antihistamine fixed-dose combination therapy	
Q6	In practice, how frequently do you monitor patients on each of the following treatments,	Columns:<br>1. Once a month or more frequently	

	in case treatment adjustment is required based on response to treatment (including no response)?	2. Once every 2 months<br>3. Once every 3 months<br>4. Once every 4 month or less frequently<br>5. N/A – I do not prescribe this treatment regimen<br><br>Rows:<br>1. Intranasal corticosteroid therapy<br>2. Oral antihistamine treatment and intranasal corticosteroid therapy<br>3. Intranasal corticosteroid/intranasal antihistamine fixed-dose combination therapy<br>4. Oral antihistamine treatment and intranasal corticosteroid/intranasal	

		antihistamine fixed-dose combination therapy	
Q7	For **how long** do you recommend keeping a patient on a treatment regimen, once their **symptoms are controlled**, before you try to **de-escalate their treatment**?<br>Please indicate a timescale for each of the following treatment regimens.	Columns:<br>1. Up to 1 month. and re-start once symptoms re-appear<br>2. 1-2 months<br>3. 2-3 months<br>4. > 3 months<br><br>Rows:<br>1. Intranasal corticosteroid therapy<br>2. Oral antihistamine treatment and intranasal corticosteroid therapy<br>3. Intranasal corticosteroid/intranasal antihistamine fixed-dose combination therapy	OPEN RESPONSE

		4. Oral antihistamine treatment and intranasal corticosteroid/intranasal antihistamine fixed-dose combination therapy	
Q8	Do you ever recommend PRN treatment (treatment only when required) for moderate – severe allergic rhinitis?	1. No, never<br>2. Yes, occasionally<br>3. Yes, frequently	SINGLE
Q9	Please explain under what circumstances / for which patients, is PRN (treatment only when required) an appropriate treatment option for patients with moderate – severe allergic rhinitis?		OPEN<br>DO NOT SHOW IF SELECT 1, NO NEVER AT Q8
Q10	Which of the following treatments would you be comfortable to prescribe on a PRN basis?<br>*Please select all that apply*	1. Oral antihistamine treatment<br>2. Intranasal corticosteroid therapy	MULTI SELECT.<br>DO NOT SHOW IF SELECT 1, NO NEVER AT Q8

		3. Intranasal corticosteroid/intranasal antihistamine fixed-dose combination therapy<br>4. Other, please specify	
Q11	At what point do you consider recommending surgery to a patient with moderate – severe allergic rhinitis?	5. Never – I do not recommend surgery for allergic rhinitis<br>6. If their only uncontrolled symptom is nasal drip<br>7. If their symptoms remain uncontrolled on 3rd line therapy<br>8. If their symptoms are temporarily controlled but frequently relapsing for 1 month<br>9. If their symptoms are temporarily controlled but frequently relapsing for 3 months	MULTI SELECT

		10. If their symptoms are temporarily controlled but frequently relapsing for 6 months<br>11. Other, please specify	
CLOSE	**That is the end of the questionnaire – thank you for your responses. This research is sponsored by GSK.**<br><br>If you have any final comments, please provide these below, otherwise, click to end the questionnaire.	OPTIONAL OPEN END	

Full questionnaire findings

First-round questionnaire

Q1: How do you currently diagnose allergic rhinitis?

Diagnostic method	Number of mentions by participants
Skin prick test/RAST	15
IgE test	13
Medical history	11
Clinical symptoms	11
Nasal fibro endoscopy	7
Physical exam	5
Family history	2

Given definition of AR: “The diagnosis of allergic rhinitis is established on the basis of an analysis of the allergic history, the nature of clinical symptoms and the results of a specific allergic examination of the patient. Criteria for establishing the AR diagnosis: 1. the presence of characteristic complaints: daily manifesting for an hour or more at least two of the following symptoms: nasal congestion, rhinorrhoea, sneezing, itching in the nasal cavity; 2. the presence of anamnestic data indicating a connection between the occurrence of complaints after contact with a suspected causal allergen; 3. the presence of other allergic diseases (allergic conjunctivitis, asthma, cross food intolerance syndrome, atopic dermatitis); 4. the presence of positive results of allergy tests. Examination, treatment and dynamic observation of a patient with AR should be carried out in parallel by doctors of two specialties: ENT and allergists-immunologists, which allows to provide an optimal integrated approach

to AR therapy. The ENT conducts a visual assessment of the nasal cavity, anterior rhinoscopy, endoscopic examination of the nasal cavity and nasopharynx, conducts differential diagnosis of AR with other diseases of the nasal cavity and paranasal sinuses, identification of complicated forms of AR, reveals the presence of anatomical abnormalities in the nasal cavity, determines the indications for surgical treatment. The allergist-immunologist determines the tactics of allergic examination, conducts skin/IgE and provocative tests, interprets the results. Allergy examination, excludes or confirms the presence of asthma, determines the feasibility of ASIT."

Q2: Once a patient has been diagnosed with allergic rhinitis, how do you group / categorise them?

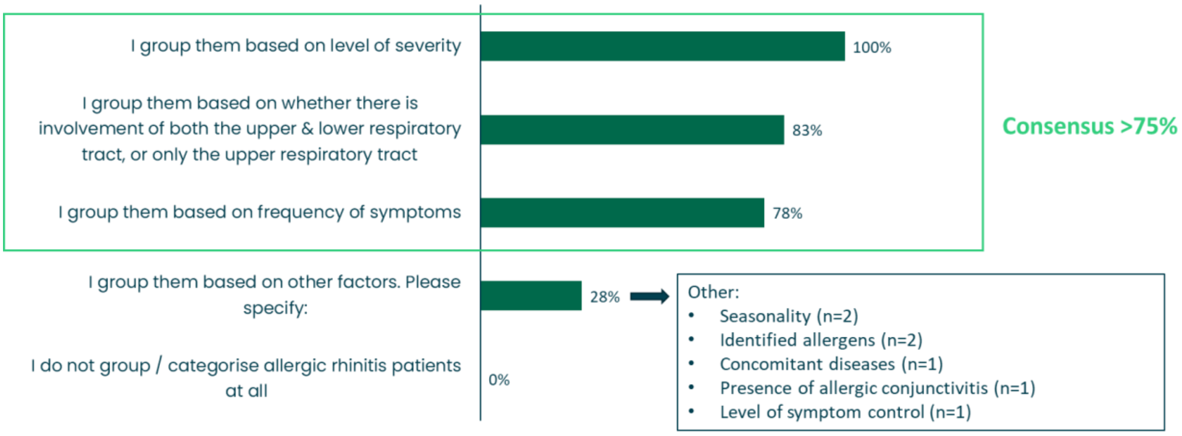

Q3: What scales do you use to determine allergic rhinitis severity (mild / moderate / severe) and / or the assessment of symptom control?

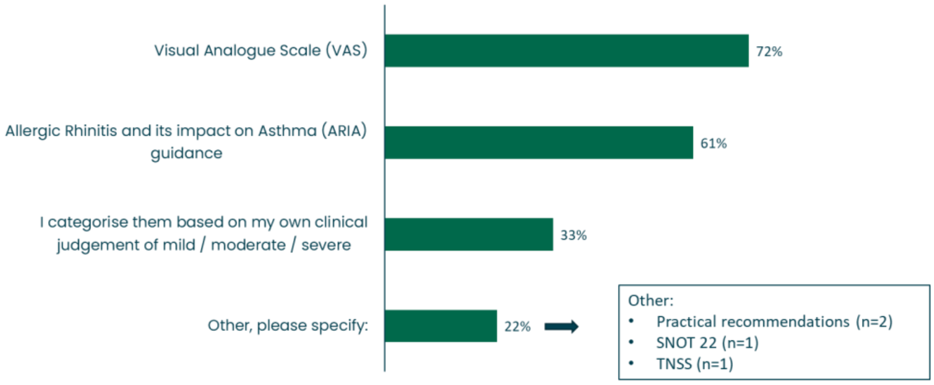

Q4: How useful do you find these scales for assessing severity of allergic rhinitis? Please rate on a scale of 1-10, where 1 is not at all useful and 10 is extremely useful.

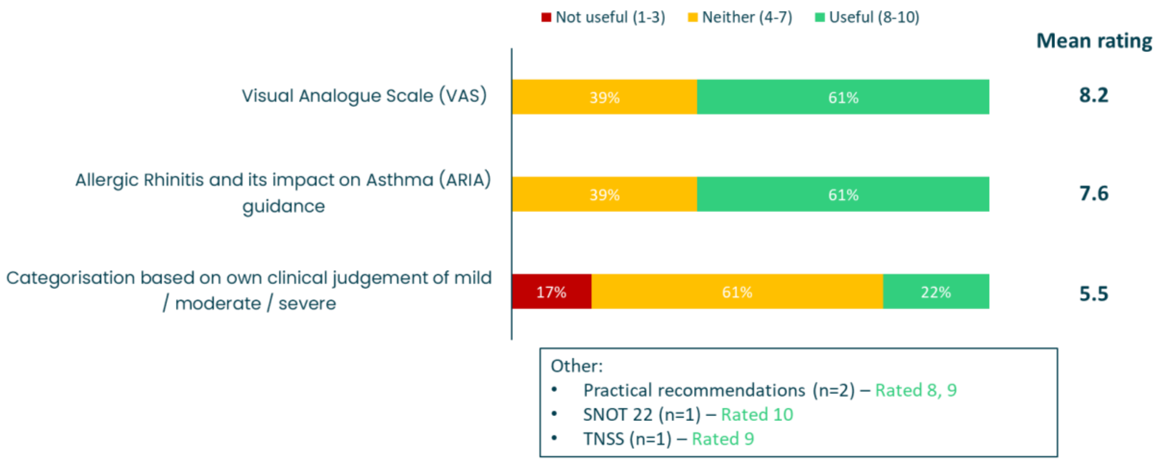

Q5: How reliable do you find these scales for assessing severity of allergic rhinitis? Please rate on a scale of 1-10, where 1 is not at all useful and 10 is extremely useful.

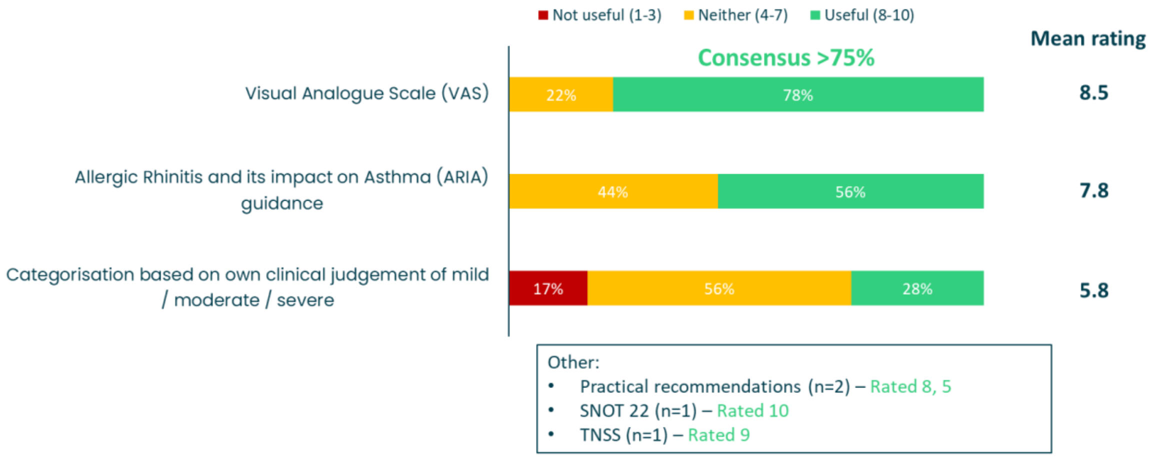

Q6: Please rank these scales in order from the easiest to use to the most difficult to use in a busy outpatient setting

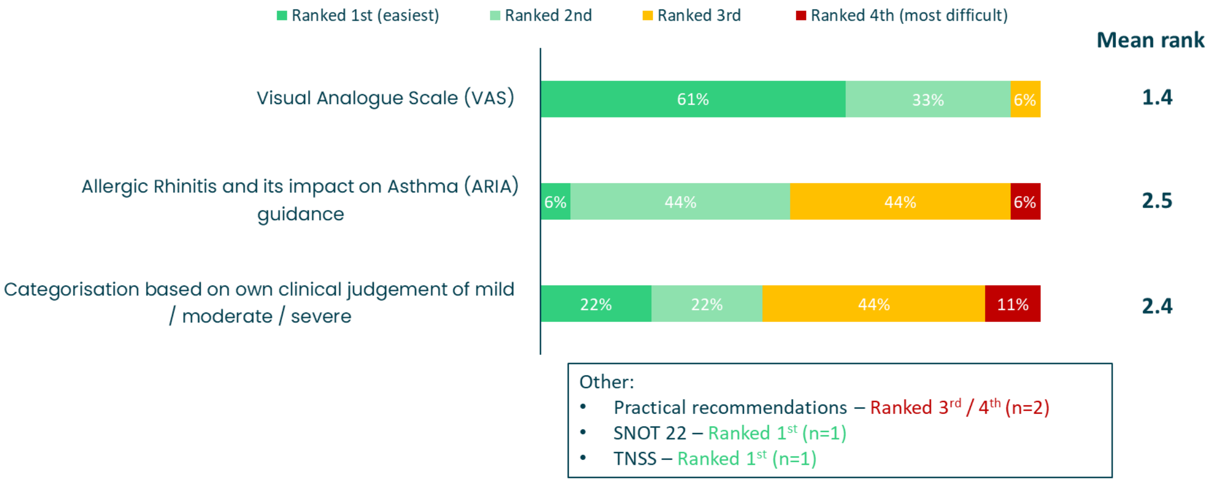

Q7: How long does it take to classify a patient's severity using these scales?

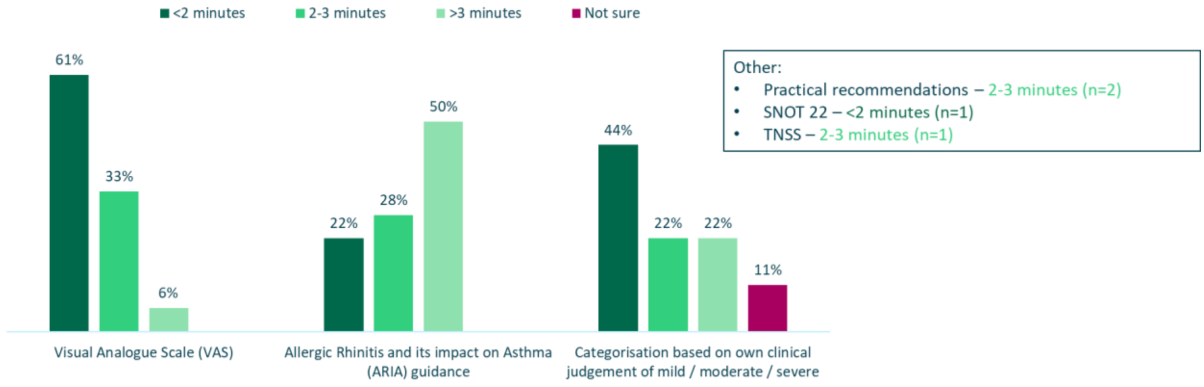

Q8: For patients with moderate-severe allergic rhinitis, who are eligible for intranasal corticosteroid but so far have only been treated with OAH for at least 3-5 days and remain uncontrolled, which of the below treatment approaches would you consider appropriate as 2nd line therapy?

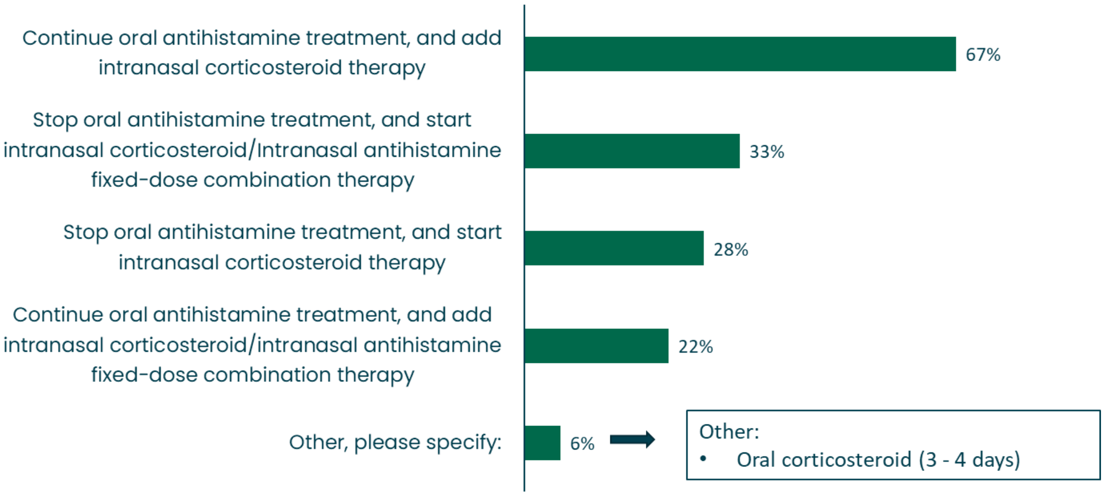

Ashfield MedComms: An Ashfield Health Company. ©2020.

Q9: Please rank the below treatment options based on their ease to implement as step-up therapy for patients with moderate-severe allergic rhinitis (1=easiest, 4=most complex)?

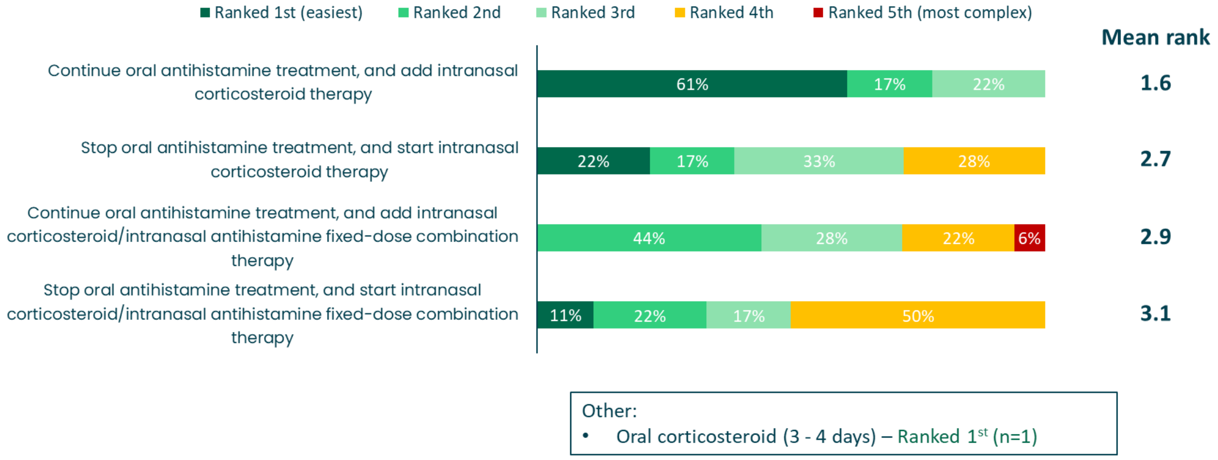

Q9a: For the treatment option you ranked as 1 (easiest to implement), please explain why you consider this to be the simplest regimen to implement for patients with moderate-severe allergic rhinitis.

**Continue OAH and add INCS (n=11)**	**Stop OAH and start INCS (n=4)**	**Stop OAH and start INCS/INAH fixed-dose combination therapy (n=2)**	**INCS + Oral corticosteroid (n=1)**
“You just add the INCS to the antihistamine. Generally, they do also have ocular symptoms and nasal histamine-induced symptoms (sneezing, pruritus, rhinorrhoea), thus that's why I do not stop the OAH they are already taking but add INCS to it. Once controlled after some weeks, depending on	“INCS have same efficacy as combination of INCS+OAH”<br><br>“In my understanding, monotherapy with intranasal corticosteroids allows to achieve and keep the control of all symptoms of allergic rhinoconjunctivitis in most patients	“Patient with moderate-Severe AR with bad response to OAH, I'd step-up with adding more drugs to OAH as INCS/AH fixed dose”	“In my opinion if the patient can use oral corticosteroid they will improve in a few days with a good result, and it is faster than others. But I like to prescribe at the same time an intranasal corticosteroid.”

symptoms they still have a bit I would continue with the one medication that best covers the bit of symptoms they still have."<br><br>"More convenient for patient"<br><br>"Prefer bridging period between OAH and INS and wait until the peak efficacy of INS"<br><br>"Patients are used to OAH and want to stay with them. Starting with intranasal steroids is easier avoiding a fixed schedule, allowing patients to understand their efficacy."	with moderate to severe AR with minimal risk of adverse effects"<br><br>"No additive effect of oral anti histamines to nasal steroid has not been certified. The mixture of nasal steroid with nasal anti-histamine is not available in Japan"<br><br>"We have the efficacy, safety and administration facility with the administration of a single therapeutic treatment."		

"Compliance with the principle of stepwise therapy, obtaining a cumulative response for OAH, impact on the early and late phases of the allergic response, quick anti-inflammatory effect. This therapy option should be considered for 2 weeks of therapy."<br><br>"It is easier to add new treatment than change all treatment"			

Q10: Please rank the below treatment options based on their efficacy for patients with moderate-severe allergic rhinitis (1=most efficacious, 4=least efficacious)?

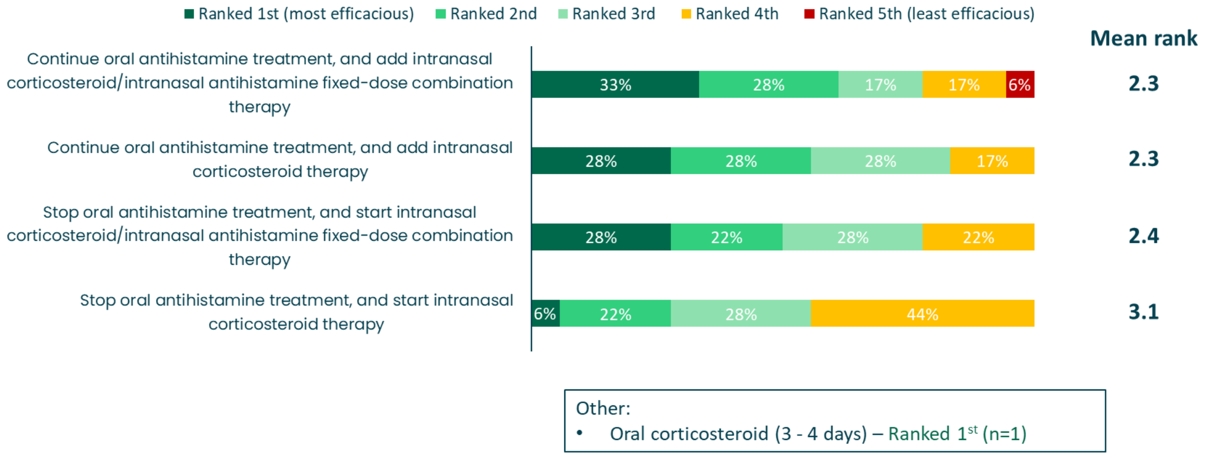

Q11: Please rank the below treatment options based on their level of dosing frequency difficulty (1=easiest dosing frequency, 4=most complex dosing frequency)?

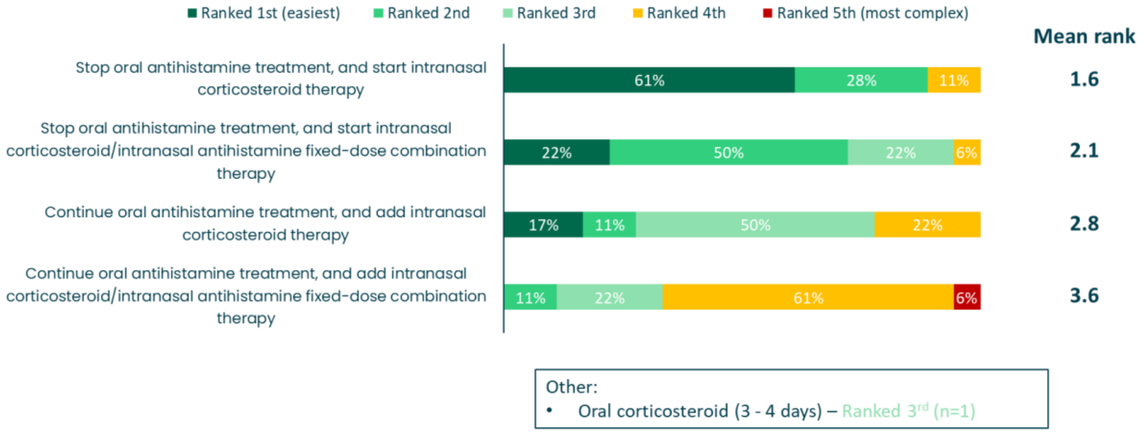

Q10a: For the treatment option you ranked as 1 (most efficacious), please explain why you consider this to be the most efficacious treatment for patients with moderate – severe allergic rhinitis

Continue OAH and add INCS/INAH fixed-dose combination therapy (n=6)	Continue OAH and add INCS (n=5)	Stop OAH and start INCS/INAH fixed-dose combination therapy (n=5)	Stop OAH and start INCS (n=1)	INCS + Oral corticosteroid (n=1)
"Good efficacy with few side effects." "The intranasal combo covers both inflammation and symptoms secondary to histamine. Also, it has better penetration into the deeper parts of the nasal cavity. Even so, some patients do not control eye symptoms (and sometimes not even nose symptoms) completely, that's	"Most of the patients are symptom controlled with INS alone but have to wait for few days to a week to let INS work thus bridging with OAH should be given during this period"	"Intranasal antihistamines work faster and improve patient's compliance" "Because the effects are reinforced" "Just based on the publications. The combined mixture is not available in Japan."	"It is useful for patients with PAR"	"Good and fast results"

why combining it with systemic OAH is even stronger therapy than monoTx"<br>"Obtaining a cumulative response for OAH, impact on the early and late phases of the allergic response, quick anti-inflammatory effect."<br>"OAH plus INCS/AH provide more control on AR symptoms"<br>"We can see some results from clinical trials where INS/AZE efficacy is better than oral antihistamines and INS as monotherapy."		"Due to fast and complete effect of intranasal combination"<br>"It has a fastest onset, and the combination of nasal steroids and intranasal antihistamines is superior to a monotherapy use with any of them."		

Q11a: For the treatment option you ranked as 1 (easiest dosing frequency), please explain why you consider this to be the best dosing regimen for patients with moderate-severe allergic rhinitis.

**Stop OAH and start INCS (n=11)**	**Stop OAH and start INCS/INAH fixed-dose combination therapy (n=4)**	**Continue OAH and add INCS (n=3)**
"Not the best, but the easiest"<br>"Two puffs each nostril twice a day, patients usually easily follow the prescription"<br>"Easy to explain"<br>"There is no need to think about the doses of drugs, the required dosages are already contained in the intranasal corticosteroid, especially if the INCS is prescribed once a day"<br>"Many intranasal corticosteroids can be used once a day."	"It is easier to use FDC"<br>"It is easier to use one route of treatment than combine intranasal spray and tablets"<br>"Fixed combination of INS/AZE is easy to use and improve adherence between patients. To use an additional oral antihistamine to that nasal spray could decrease adherence in the long term treatment."	"Patients are used to OAH and want to stay with them. Starting with intranasal steroids is easier avoiding a fixed schedule, allowing patients to understand their efficacy.

“In my experience, the intranasal regimen is easier for the patient to dose than the one combined with the oral route.”<br>“The patients are used to use intranasal corticosteroid, so it is an easier treatment for them, twice a day and they feel that improve the symptoms”<br>“Because is a single medication, you can apply it once a day and the device is easier to use for patient.”		

Q12: Please rank the below treatment options based on their difficulty level in terms of patient compliance (1=fewest problems with patient compliance, 4=most problems with patient compliance)?

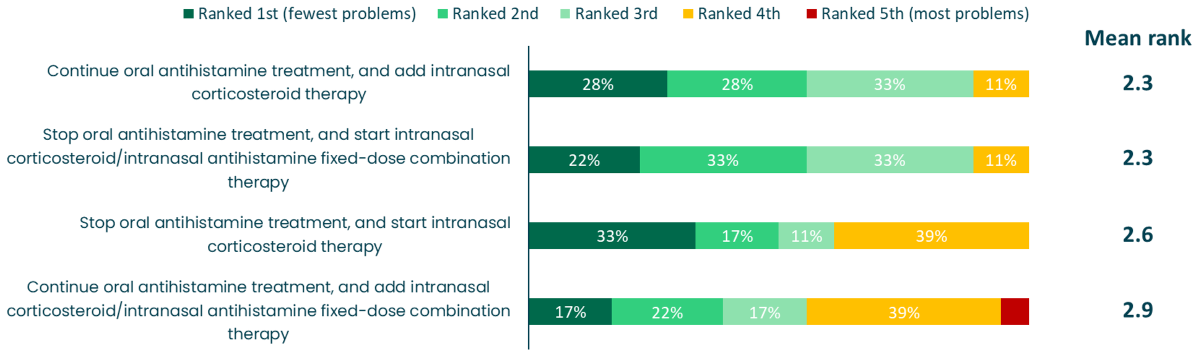

Other:
- Oral corticosteroid (3 - 4 days) – Ranked 3rd (n=1)

Q13: Please rank the below treatment options based on their safety/tolerability for patients with moderate-severe allergic rhinitis (1=most safe, 4=least safe)?

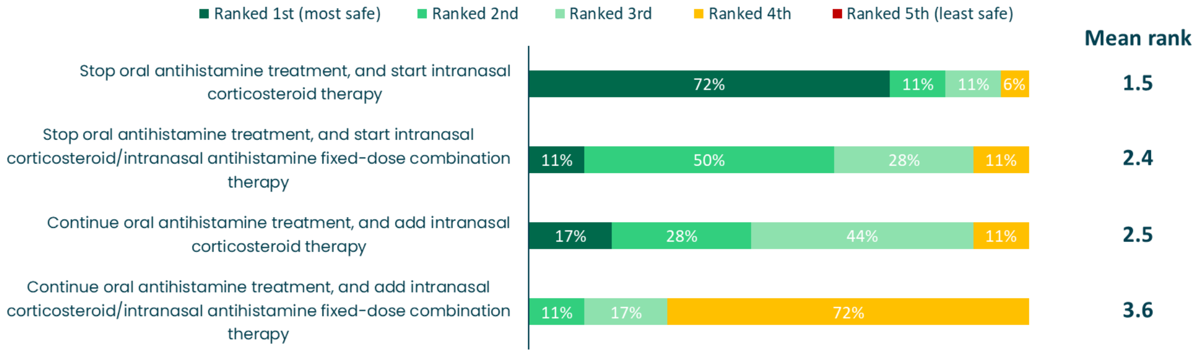

Other:
- Oral corticosteroid (3 - 4 days) – Ranked 5th (n=1)

Q12a: For the treatment option you ranked as 1 (fewest problems with patient compliance), please explain why you consider this to be the best option for patient compliance.

**Stop OAH and start INCS (n=6)**	**Continue OAH and add INCS (n=5)**	**Stop OAH and start INCS/INAH fixed-dose combination therapy (n=4)**	**Continue OAH and add INCS/INAH fixed-dose combination therapy (n=3)**
• "Few side effects"<br>• "INCS do not have bitter taste, thus adherence is better; several INCS are also a bit cheaper than INCS+AZE"	• "Patients are used to OAH and want to stay with them, while starting with intranasal steroids. This combination will help patient compliance."<br>• "Because the administration is once	• "Because only one device is used"<br>• "It is efficient that is important for patient's compliance"<br>• "All patients prefer easier treatment with fast efficacy"	• "Easy to follow the prescription"<br>• "The better the effect, the better the adherence, especially during the first two weeks of AR treatment, but with long-term therapy, the patient can assess the tolerance, the presence or absence of symptoms and stop therapy on his own. Each

• "Again, easy to explain with use less med as possible seems to be more practical"<br>• "Because It's an easier and feasible treatment to do every day."	a day, it's easier because of the device and frequency is well tolerated by the patient."	• "The fewer medications, the better compliance to the treatment for chronic diseases. Fixed combination of an INS/AZE and INS as monotherapy have demonstrated good adherence"	patient is individualized, the therapy option should be carried out together with the patient"

Q13a: For the treatment option you ranked as 1 (most safe), please explain why you consider this to be the safest treatment for patients with moderate – severe allergic rhinitis.

**Stop OAH and start INCS**<br><br>**(n=13)**	**Stop OAH and start INCS/INAH fixed-dose combination therapy**<br><br>**(n=3)**	**Continue OAH and add INCS**<br><br>**(n=2)**
• "The safest way of starting with intranasal steroids will be avoiding their association to OAH."<br><br>• "Antihistamines can cause drowsiness and other side effects when taken orally or intranasally, as well as a bitter taste can prevent intranasal corticosteroid/intranasal antihistamine fixed-dose combination from being used for longer than 1-2 weeks"<br><br>• "The monotherapy with intranasal corticosteroids allows to achieve and keep the control of all symptoms of	• "Intranasal corticosteroids are extremely safe in the recommended doses, and adherence to treatment is very high"<br><br>• "Intranasal combination with fixed doses is the safest treatment for AR patients"	• "Oral new generation antihistamines have great safety profiles; however, some minor adverse events have been reported. The fixed combination

allergic rhinoconjunctivitis in most patients with moderate to severe AR with minimal risk of adverse effects."<br>• "In general, the intranasal route has fewer side effects or adverse events than the oral route."<br>• "They are many articles showing that intranasal corticosteroid is a safer medication to be used for long time."<br>• "Only one treatment, less possibilities for side effects."<br>• "Because the newest intranasal steroids has a very low bioavailability, and the adverse effects are very rare."		on INS/AZE have also some local adverse events as dryness, aftertaste or usually mild epistaxis.<br>• "There is no evidence of ADR from combination OAH, INS fixed combination INS/TAH get intolerable effects esp. from the taste"

Q14: Which of the below treatment regimens, once prescribed, can continue for 3 months or until allergy season, without interim review or adjustment if the patient remains controlled?

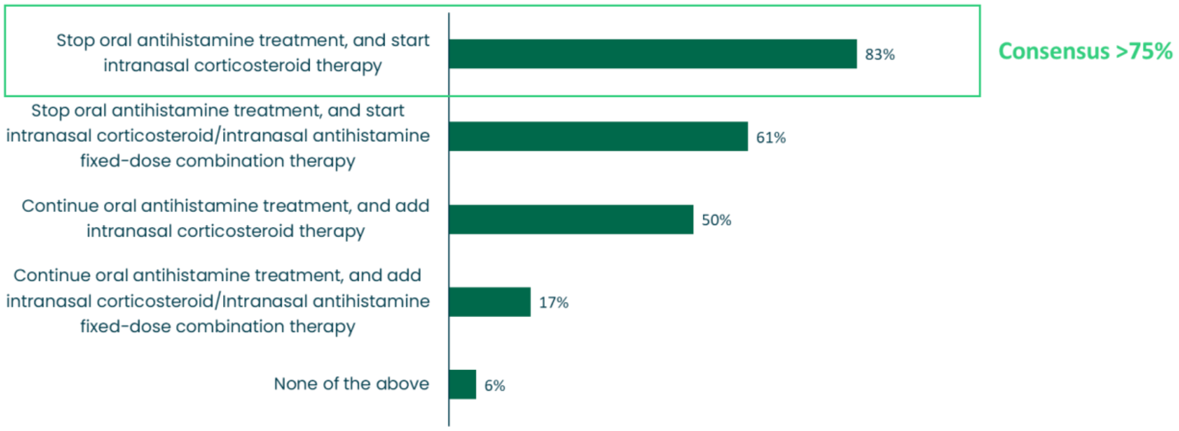

Q15: Which of the above treatment regimens, once prescribed, should be monitored monthly or more frequently, in case treatment adjustment is required, based on response to treatment (including no response)?

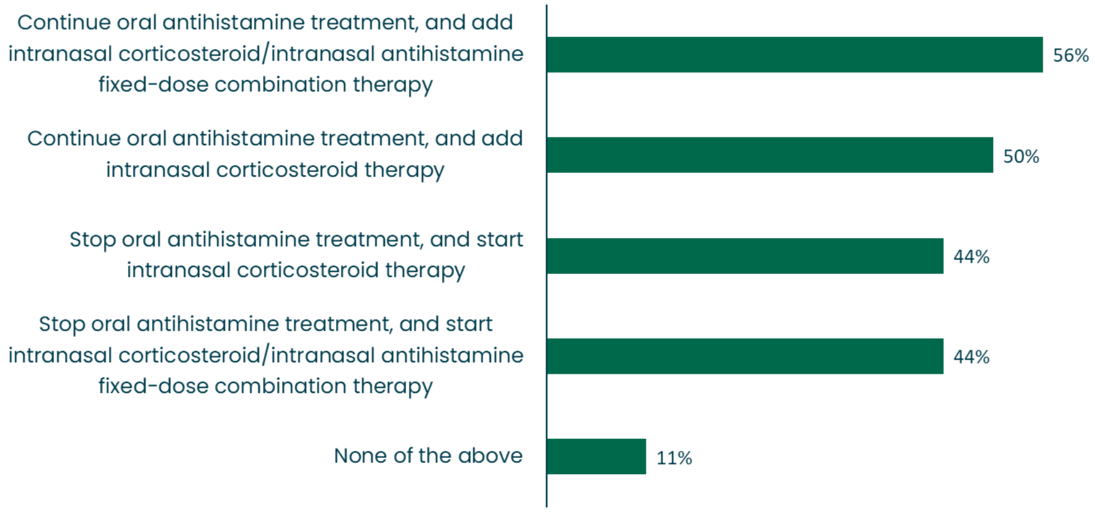

Q16: Do you use allergen immunotherapy in patients with AR?

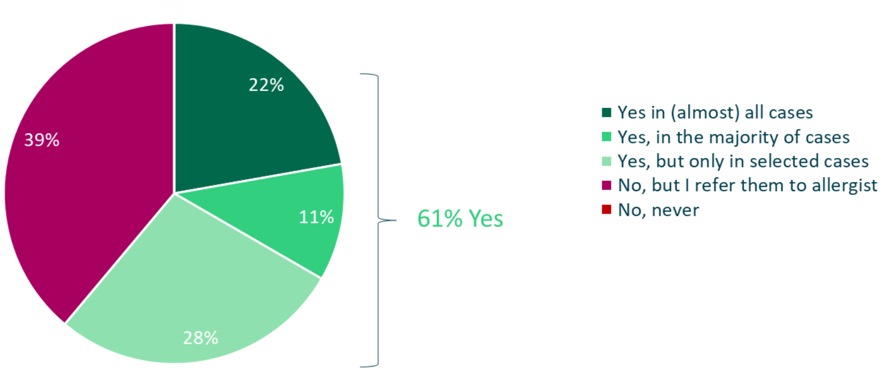

Wave 2 questionnaire

Q1: To what extent do you agree or disagree with the following statements?

Diagnosis of allergic rhinitis is best achieved through a combination of clinical observation / patient history, skin prick tests and IgE tests. Diagnosis of allergic rhinitis should be made through a collaborative approach between ENT specialists and allergists, where necessary to ensure access to allergy testing

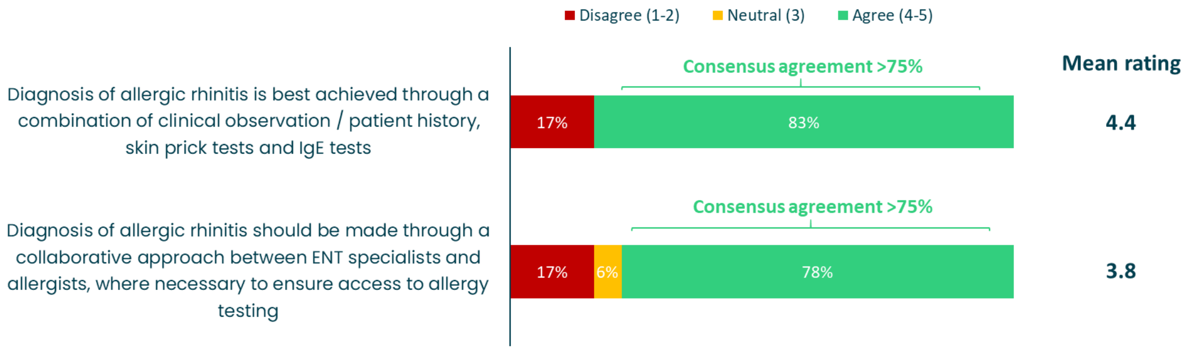

Q2: Please think about how you assess the severity and/or symptom control of a patient diagnosed with allergic rhinitis. Taking into account all factors, including usefulness, reliability and ease of use, which scale do you think is the best assessment to use in clinical practice?

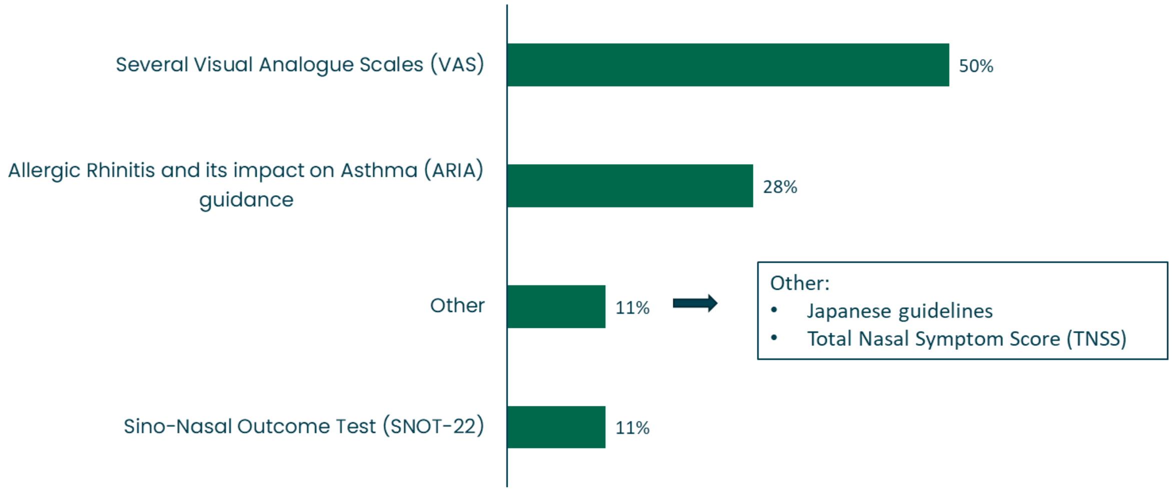

Q3: How long does it take to classify a single patient's severity using these scales?

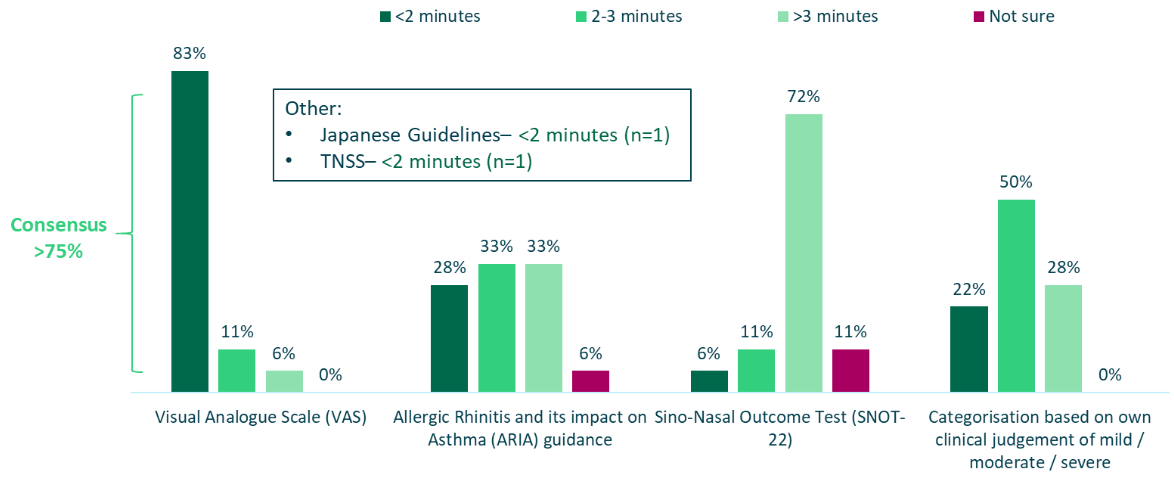

Q4: For patients with moderate-severe allergic rhinitis, who are eligible for intranasal corticosteroid but so far have only been treated with oral antihistamine for 3-5 days and remain uncontrolled, which of the below treatment approaches would you consider to be the most appropriate 2nd line therapy?

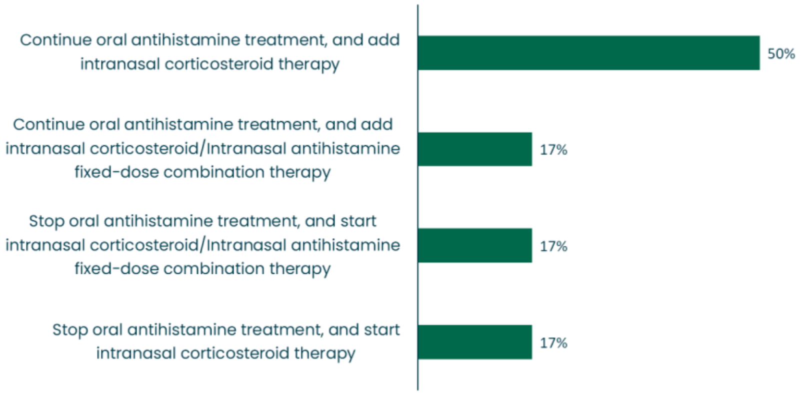

Q5: Please select which of the below treatment options is the most favourable based on each of the following factors:

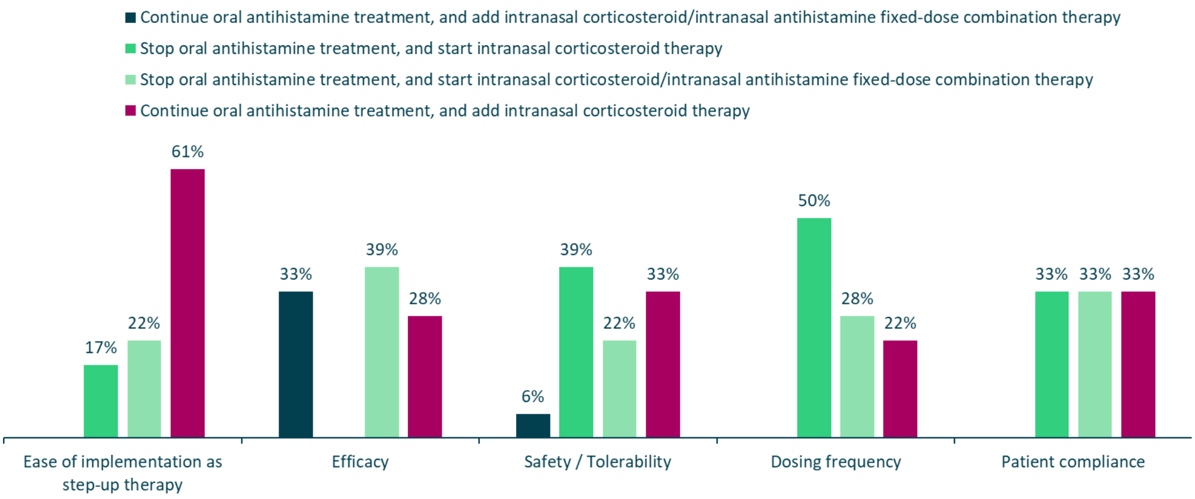

Q6: In practice, how frequently do you monitor patients on each of the following treatments, in case treatment adjustment is required based on response to treatment (including no response)?

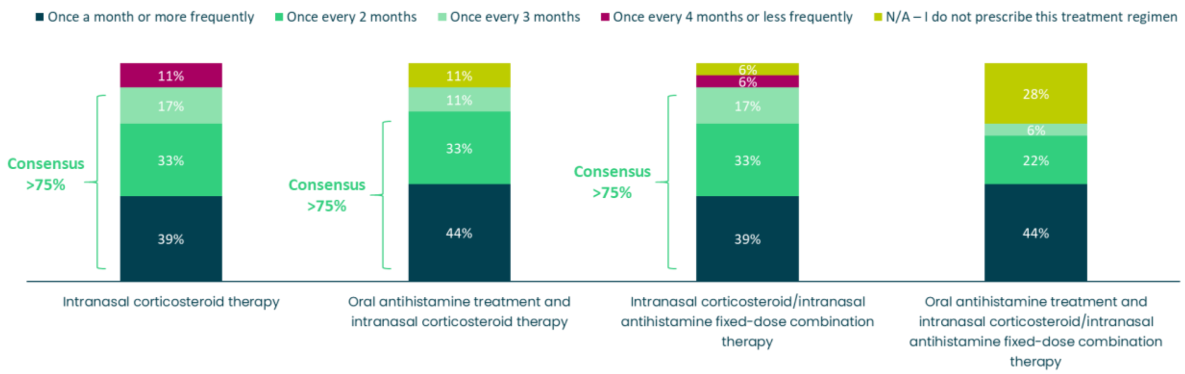

Q7: For how long do you recommend keeping a patient on a treatment regimen, once their symptoms are controlled, before you try to de-escalate their treatment? Please indicate a timescale for each of the following treatment regimens.

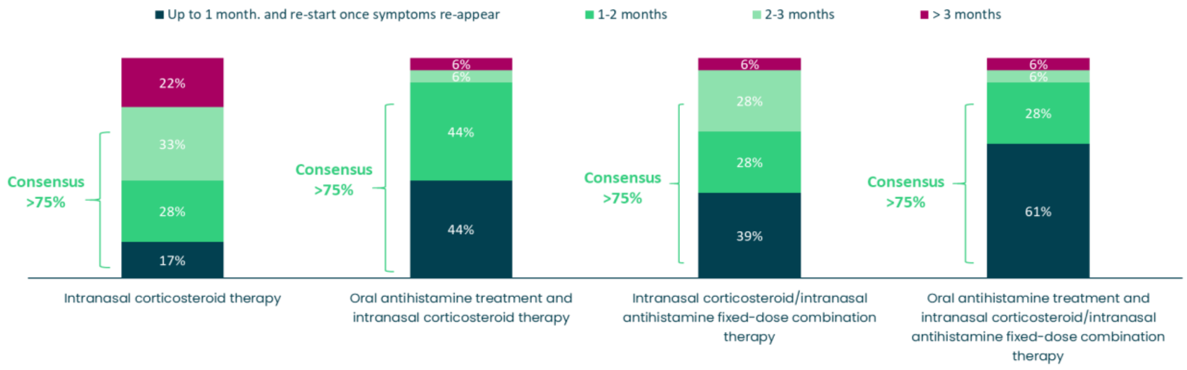

Q8: Do you ever recommend PRN treatment (treatment only when required) for moderate – severe allergic rhinitis?

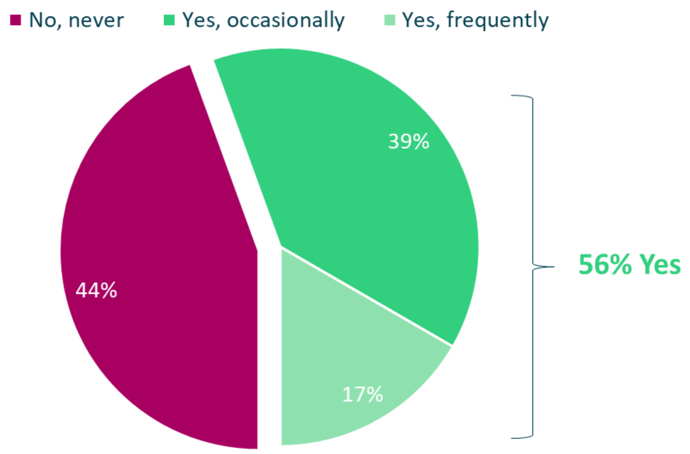

Q9: Please explain under what circumstances / for which patients, is PRN (treatment only when required) an appropriate treatment option for patients with moderate – severe allergic rhinitis?

**CIRCUMSTANCES FOR PRN TREATMENT (n=10)**
“Applicable in the case of intermittent allergic rhinitis in adults in the absence of constant contact with the allergen.”
“Out of seasonal AR and in case of some mild adverse effect of treatment (intranasal treatment).”
“Seasonal allergic rhinitis. In combination with immunotherapy”
“Normally I use long term and daily treatments, however in real life when the patients improve, they usually stop the recommended treatment, and they decide to use it when the symptoms recur. According to this, a controlled patient in their symptoms I suggest them to use the medication as needed. Above all, in patients that do not have

good compliance with medications, I use short term medications and then suggest to them PRN medications."
"If control of symptoms is achieved, I would recommend using medication PRN"
"We can use PRN when the patients have symptoms such as sneezing, scratching, nasal obstruction and headache after a specific situation, such as dust exposure and temperature change."
"When patients are able to remain for at least 1 month with the symptoms controlled without medications."
"Once the patient is completely controlled and out-of-season, still PRN use of INCS is OK, to keep patients from relapsing too badly when they are again exposed to one of their allergens. They normally do it already themselves. If they are OK, start using the INCS only PRN."
"When compliance is expected to be very low."
"If the patient has symptoms"

Q10: Which of the following treatments would you be comfortable to prescribe on a PRN (treatment only when required) basis?

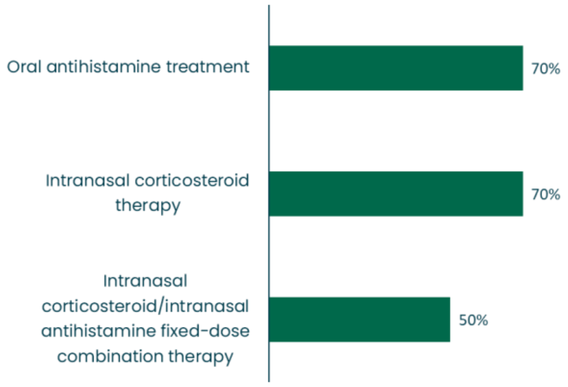

Q11: At what point do you consider recommending surgery to a patient with moderate – severe allergic rhinitis?

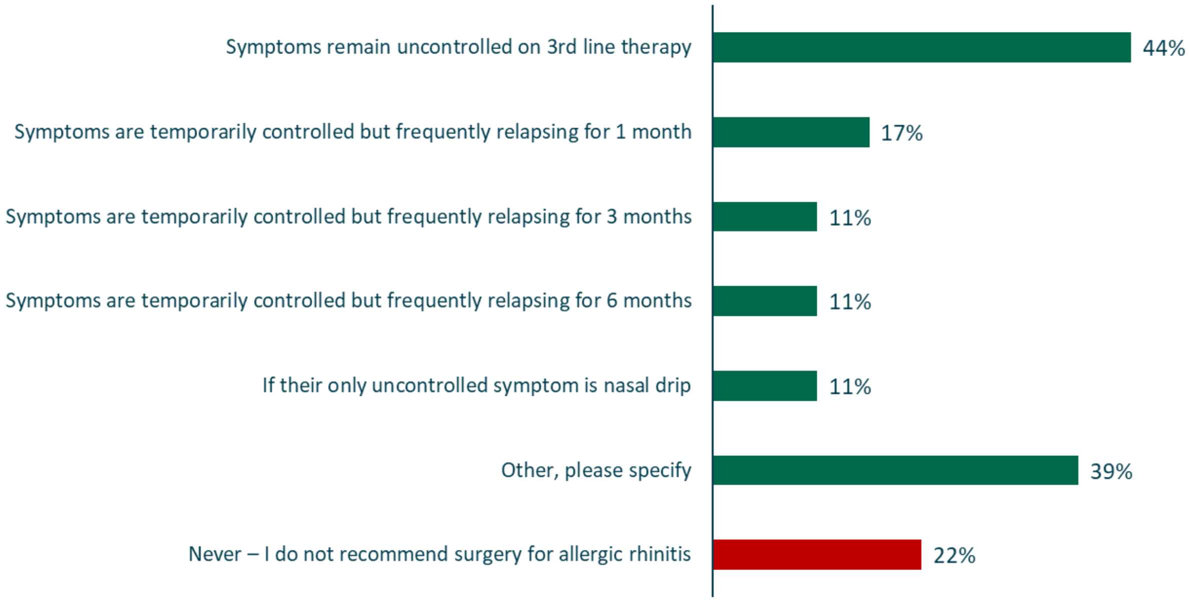

**Other:**
"Deviated nasal septum and other anatomical abnormalities, rhinitis medicamentosa"
"Turbinate RF is recommended based on clinical findings and unresponsiveness to treatment and local decongestive."
"Surgical intervention in AR is performed only if the patient has a comorbidity. The indication for surgery in patients with AR with nasal obstruction is determined by the ENT based on the results of clinical (including endoscopic examination of the nasal cavity and nasopharynx) and radiological examination of the patient"
"I really don't believe that surgery can figure out it. But sometimes we can have "meeting" between ENT and allergist to discuss the best alternative for some

cases. And if the surgery was indicated we have to explain the patient that this is an alternative not absolute solution”
“It’s not only the doctor’s decision, but the patient also needs to be aware of the risk: benefit ratio and agree with the surgery indication.”
“If the nasal blockage is just not well controlled or unilateral”
